# Metabolic folate profiling as a function of time during cultivation suggests potential C2-metabolism in *Saccharomyces cerevisiae*

**DOI:** 10.3389/fnut.2022.984094

**Published:** 2022-10-19

**Authors:** Lena Schillert, Daniela Wirtz, Nadine Weber, Franziska Schaller, Lisa Striegel, Philippe Schmitt-Kopplin, Michael Rychlik

**Affiliations:** ^1^Chair for Analytical Food Chemistry, Technical University of Munich, Munich, Germany; ^2^Research Unit BioGeoChemistry, Helmholtz Zentrum Munich, Neuherberg, Germany; ^3^Queensland Alliance for Agriculture and Food Innovation, The University of Queensland, Coopers Plains, QLD, Australia

**Keywords:** folates, polyglutamates, Baker's yeast, C2-metabolites, EthylFox, FT-ICR-MS, foodomics, metabolomics

## Abstract

Yeasts are reported to be rich in folates, a group of vitamers known to be involved in several biosynthetic reactions such as methylation reactions, oxidation and reduction processes, and nucleotide synthesis. Not being able to synthesize folates, humans rely on external folate supply. Here, we show the application of LC/MS-MS methods using SIDA (stable isotope dilution analysis) assays for the quantitative analysis of different folate mono- and polyglutamates during growth of *Saccharomyces cerevisiae*. Molecular networking (MN) was applied for detailed analysis of further folate metabolites. Highest folate contents of 13,120 μg/100 g were observed after 20 h of cultivation. The main vitamers 5-CH_3_-H_4_folate and H_4_folate decreased during cultivation, while 5-CHO-H_4_folate increased during cultivation. The hexa- and heptaglutamate of 5-CH_3_-H_4_folate accounted for >96% of the total 5-CH_3_-H_4_folate content. A shift of the major polyglutamate from hexa- to heptaglutamate was observed after 29 h. MN unraveled two groups of novel folates which could be assigned to a potentially existing C_2_-metabolism in yeast. In detail, 5,10-ethenyl-tetrahydrofolate and a further CO-substituted 5-CH_3_-H_4_folate were identified as hexa- and heptaglutamates. The latter was neither identified as 5-acetyl-tetrahydrofolate nor as EthylFox, the oxidation product of 5-ethyl-tetrahydrofolate. The structure needs to be elucidated in future studies.

## Introduction

Folates are a group of more than 150 structurally similar compounds, their entirety giving the vitamer class B_9_. The most stable form of these vitamers is the fully oxidized pteroyl glutamic acid (PteGlu), commonly known as folic acid. The common structural element of all compounds is given by the core structure consisting of a 4-amino-4-oxo-pteridine ring being linked to *p*-amino benzoic acid and one to fourteen γ-linked glutamic acid moieties (1–3). Different oxidation states within the pteridine ring, variation within the substitution at positions N^5^ and N^10^ (typically C_1_-units in the form of methyl, formyl, or methenyl groups), and varying length of the glutamyl tail result in the large number of folates known so far.

**Figure 1:**
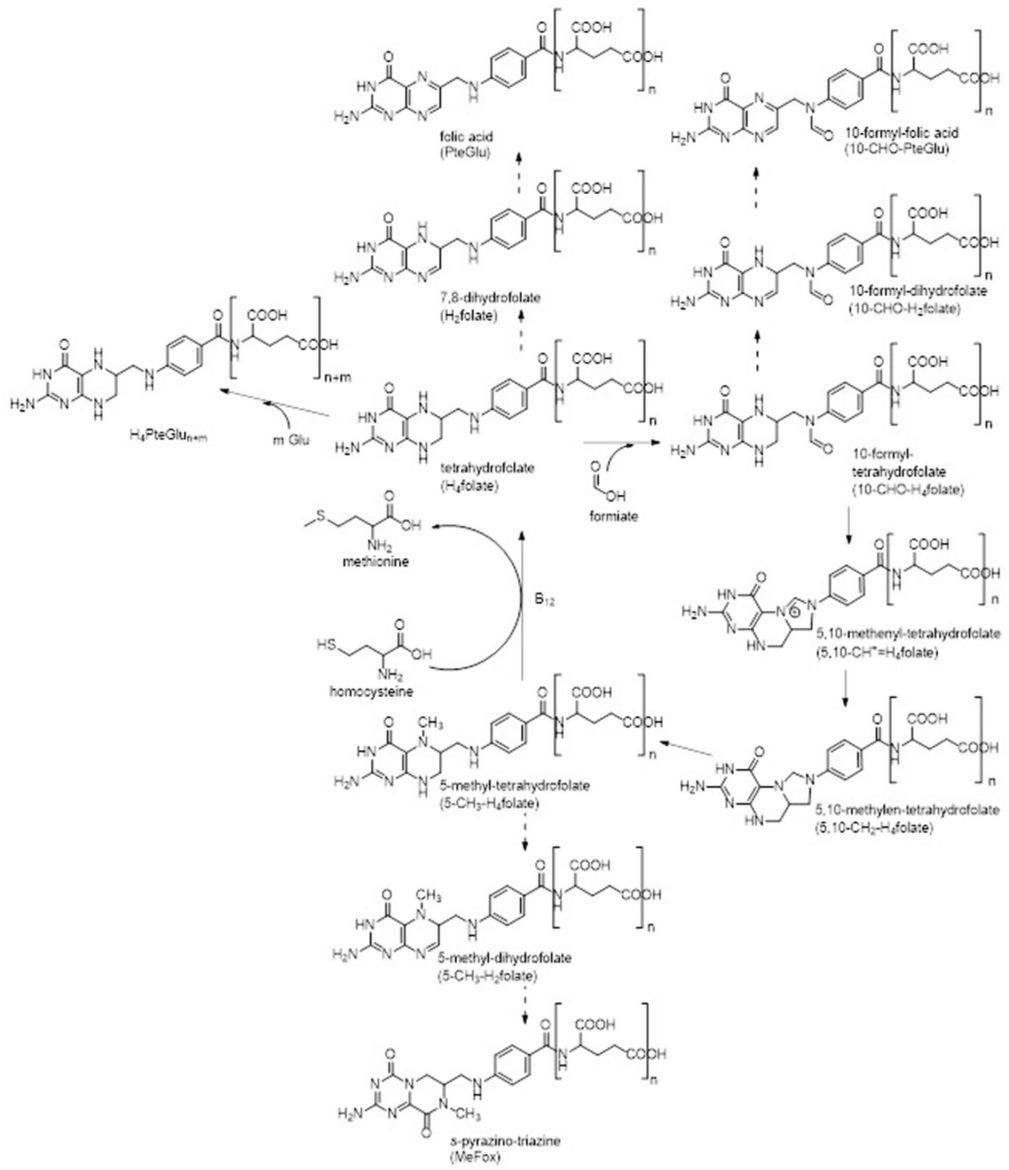
C_1_-metabolism of folate vitamers known so far. Common oxidation reactions (dashed arrows) were incorporated into the figure as well as enzymatic polyglutamylation. Enzymes participated in interconversion reactions are not stated for the sake of clarity.

Generally, folates serve as cofactors in the transfer of C_1_-units in a variety of biochemical reactions within the organism (4–6). Such reactions are for instance purine and thymidylate synthesis and thus DNA replication as well as methylation reactions (1, 3). A part of this C_1_-metabolism is shown in Figure 1 in which the metabolic active 5-methyl-tetrahydrofolate (5-CH_3_-H_4_folate) serves as methyl donor for the reactivation of methionine by methylation of homocysteine (7). A lack of folates has been linked to several chronic diseases such as neurogenerative disease, cardiovascular diseases, or Alzheimer's disease (8–10). Most severe, however, is the incidence of neural tube defects in newborns as result of a maternal malnutrition with folates during pregnancy (11). Yet mammals are not able to synthesize folates *de novo* and thus rely on a sufficient external supply. Yeast has been proven to be a good folate source due to high folate contents above 4,000 μg/100 g.

To date, folate analysis mostly focused on quantitative analysis of different vitamers after enzymatic deconjugation into the respective monoglutamates (1, 3, 12). Yet little is known about the folate metabolism especially with regards to polyglutamates which has been shown to account for the majority of the folate pool in organisms (13–15). Consequently, there is a clear need for deeper research on the metabolism of folates as a whole.

In a previous study we could show that high resolution mass spectrometry (HR-MS) applying quadrupole time of flight MS (Q-ToF-MS) provides a more profound tool for folate metabolic profiling (16). However, folates are only rarely included into one of the numerous spectral databases available (a manual search in a selection of databases was applied). If at all, only spectral information for monoglutamates is accessible which makes a comparison of experimental data rather challenging. Therefore, other tools for data analysis need to be considered. A promising tool for this purpose is molecular networking (MN). MN offers the possibility for undirected analysis of fragmentation information of an experimental setup. Global Natural Products Social Molecular Networking (GNPS) offers a web-based application of MN which is freely available (17). GNPS uses the idea of clustering nearly identical tandem-MS spectra in one consensus spectrum (18, 19). Those consensus spectra are matched against each other to find similarities within these spectra, assuming that structurally similar molecules show similar fragmentation behavior in MS/MS analysis. Spectra exceeding the user-defined similarity threshold will subsequently be visualized within one cluster “family.” In those clusters, consensus MS/MS spectra are represented by nodes. Significant similarities above the chosen match score are visualized by connecting edges.

In this study we show the application of MN for a deeper analysis of folate metabolites. *S. cerevisiae* was chosen as model organism due to its reportedly high folate contents of >4,000 μg/100 g (20, 21) and the possibility for cultivation according to our needs. The chosen yeast strain was cultivated in minimal medium with varying length of cultivation time to trace the development of the folate composition during yeast growth. Samples were quantitatively analyzed for their mono-and polyglutamate content by means of LC-MS/MS (liquid chromatography tandem mass spectrometry) to show the development of different monoglutamates and 5-CH_3_-H_4_folate polyglutamates during cultivation. Furthermore, samples were analyzed by means of Q-ToF-MS for further analysis of folate polyglutamates and subsequent application of MN. MN was applied to give deeper insight into the folate metabolism in yeasts and to uncover potential further folate vitamers with focus on the polyglutamates.

## Materials and methods

### Chemicals and materials

Unless otherwise stated, solvents and chemicals were of analytical grade. Methanol (MeOH), acetonitrile (ACN), and formic acid were of LC-MS grade (Sigma-Aldrich, Germany). Water was derived from a Merck Millipore Integral 3 water purification system (Billerica, MA, USA). Folate standards and internal standards were purchased form Schircks Laboratories (Jona, Switzerland). Only MeFox was purchased from Merck & Cie (Schaffhausen, Switzerland). Chicken pancreas and rat serum were obtained from Biozol (Eching, Germany) and Difco (Sparks, MD, USA). Strata strong anion exchange (SAX) cartridges (quaternary amine, 500 mg, 3 mL, 1 g, 6 mL, and 2 g, 12 mL) were purchased from Phenomenex (Aschaffenburg, Germany); Chromabond hydrophilic lipophilic balanced (HLB) cartridges (60 μm, 500 mg, 3 mL) were obtained from Macherey-Nagel (Düren, Germany).

### Yeast strains and cultivation

The top-fermenting brewer's yeast *Saccharomyces cerevisiae* LeoBavaricus TUM68^®^ was provided by the Yeast Center at the Weihenstephan Research Center for Brewing and Food Quality of the Technical University of Munich. Yeast cultivation and the sampling procedure are described in detail in the Supplementary material. Baker's yeast for matrix matched calibration functions was bought in a local supermarket (Freising, Germany), freeze-dried, homogenized using a pestle and mortar, and stored at −20°C until usage.

### Sample extraction, analysis, and data preprocessing for folate profiling by UHPLC-Q-ToF-MS

Sample preparation was performed under subdued light. Sample extraction of 450 mg of lyophilized yeast sample was as described in a previous publication for high resolution measurements by ultra-high pressure liquid chromatography (UHPLC) Q-ToF-MS (16). Samples were spiked with the internal standard (ISTD) [^13^C_5_]-5-CH_3_-H_4_folate right before injection into the UHPLC-Q-ToF-MS system. Parameters used for analysis can be found in the Supplementary material. Data preprocessing of UHPLC-Q-ToF-MS data was as in the above mentioned publication. Within the corresponding retention time window of folates, MS-features were run against an inhouse database to unravel putative folate vitamers. Identification was achieved by comparison of the respective tandem-MS data.

### Quantification of the total folate content and of 5-CH_3_-H_4_PteGlu_1 − 7_

Detailed information about the folate stable isotope dilution assays applied for the quantification of the total folate content can be found elsewhere (16, 22). The content of 5-CH_3_-H_4_PteGlu_1 − 7_ was quantified by a modified method also using the internal standard (ISTD) [^13^C_5_]-5-CH_3_-H_4_folate as outlined in the Supplementary material.

### Molecular networking

GNPS molecular networking parameters were set to a minimum requirement of five ions match, a minimum cluster size of 2 and a cosine score of >0.6 (https://gnps.ucsd.edu). Precursor ion and fragment ion mass tolerance were set to 0.02 Da. To further putatively identify folate metabolites in the yeast samples analyzed, mass spectral molecular networking was applied with a preliminary dataset analyzed by UHPLC-Q-ToF-MS. We then extracted the *m/z* of compounds clustering within the folate molecular cluster into measurements of the complete dataset by adapting the preference list for fragmentation accordingly (Supplementary Table 2). We then performed a second iteration of MN with the whole dataset. Different cultivation times were processed individually to receive independent networks for each time point. The obtained folate clusters were exported and further analyzed using RStudio software. Precursor masses of the folate clusters obtained by GNPS were compared with an inhouse database containing several folate vitamers. Annotation was verified with an annotation error of <10 ppm. Unknown features within the networks were manually reanalyzed in Compass DataAnalysis 4.3 (Bruker Daltonics, Bremen, Germany) and common features were searched.

### Software and statistics

OriginPro 2020 (OriginLab Corporation, MA, USA) and Cytoscape 3.7.2 (cytoscape.org) were used for the generation of plots. Compass DataAnalysis 4.3 (Bruker Daltonics, Bremen, Germany) and RStudio software version 3.6.1 (Boston, MA, USA) were used for data processing. OriginPro 2020 was used for statistical evaluation.

## Results

### Total folate contents

**Figure 2:**
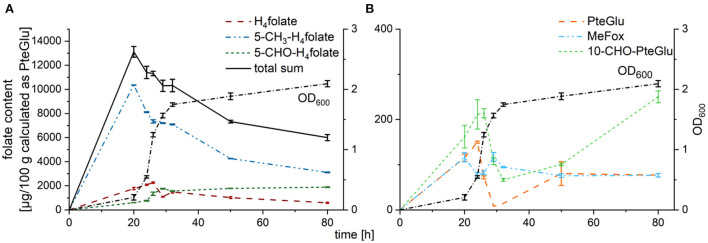
Folate contents of different vitamers in TUM68^®^ yeast during cultivation after enzymatic deconjugation, calculated as μg/100 g PteGlu (dry weight, d.w.); with **(A)** main vitamers H_4_folate (red), 5-CH_3_-H_4_folate (blue), 5-CHO-H_4_folate (darkgreen), and the total sum (black) in comparison to the optical density (OD_600_, dotted line), **(B)** oxidation products PteGlu (orange), MeFox (lightblue), and 10-CHO-PteGlu (leafgreen) in comparison to the optical density (OD_600_, dotted line). Data are expressed as mean values ± confidence limits.

Total folate analysis by LC-MS/MS revealed decreasing folate contents in cultured yeast with increasing cultivation time as shown in Figure 2. Folate was not released into the culture medium as no significant amount of different folate vitamers could be observed in the culture media (data not shown). Pairwise *t*-tests were performed to evaluate significant differences between folate contents in dependence on the length of cultivation. Total folate contents were significantly different (*p* < 0.05) between the different timepoints tested except for 20 h of cultivation compared to 24 h and 24 h compared to 26 h. The 32 h timepoint did not show any significant differences compared to all the other timepoints tested expect for a cultivation time of 20 h. Highest folate contents could be observed after harvest at 20 h with a total sum of 13,119 ± 444 μg/100 g, whereas lowest contents were reached after harvest at 80 h with a total sum of 5,995 ± 253 μg/100 g. Main vitamers observable were 5-methyl-tetrahydrofolate (5-CH_3_-H_4_folate), 5-formyl-tetrahydrofolate (5-CHO-H_4_folate) representing the sum of all formyl vitamers, and tetrahydrofolate (H_4_folate) as depicted in Figure 2A. While 5-CH_3_-H_4_folate contents decreased from the first harvest timepoint on (79% of the total content at 20 h compared to 52% of the total content after 80 h), 5-CHO-H_4_folate showed clearly increasing tendency with 5% of the total sum after 20 h and 31% after 80 h. H_4_folate, however, increased from 13% (20 h) to 20% (26 h), before a decrease to 10% after 80 h could be determined. The oxidation products folic acid (PteGlu), MeFox, and 10-formyl-folic acid (10-CHO-PteGlu) (shown in Figure 2B) generally accounted for < 2% of the total content highlighting the relative stability of the folate vitamers in yeast during cultivation. Solely 10-CHO-PteGlu reached > 4% after 80 h. The degradation product pABG (*para*-aminobenzoic acid) could not be quantified with the applied method. However, profiling measurements by UHPLC-Q-ToF-MS did not show any spectra with MS^1^ features relating to pABG – neither as monoglutamate nor as polyglutamate with a varying length of the polyglutamate tail. Trends of folate contents of the oxidized forms were in accordance with tendencies observable for the corresponding reduced forms (PteGlu replicated the tendency of H_4_folate and 10-CHO-PteGlu replicated the tendency of 5-CHO-H_4_folate). Only MeFox showed a slightly different course during cultivation compared to 5-CH_3_-H_4_folate.

### 5-CH_3_-H_4_PteGlu_n_ contents

**Figure 3:**
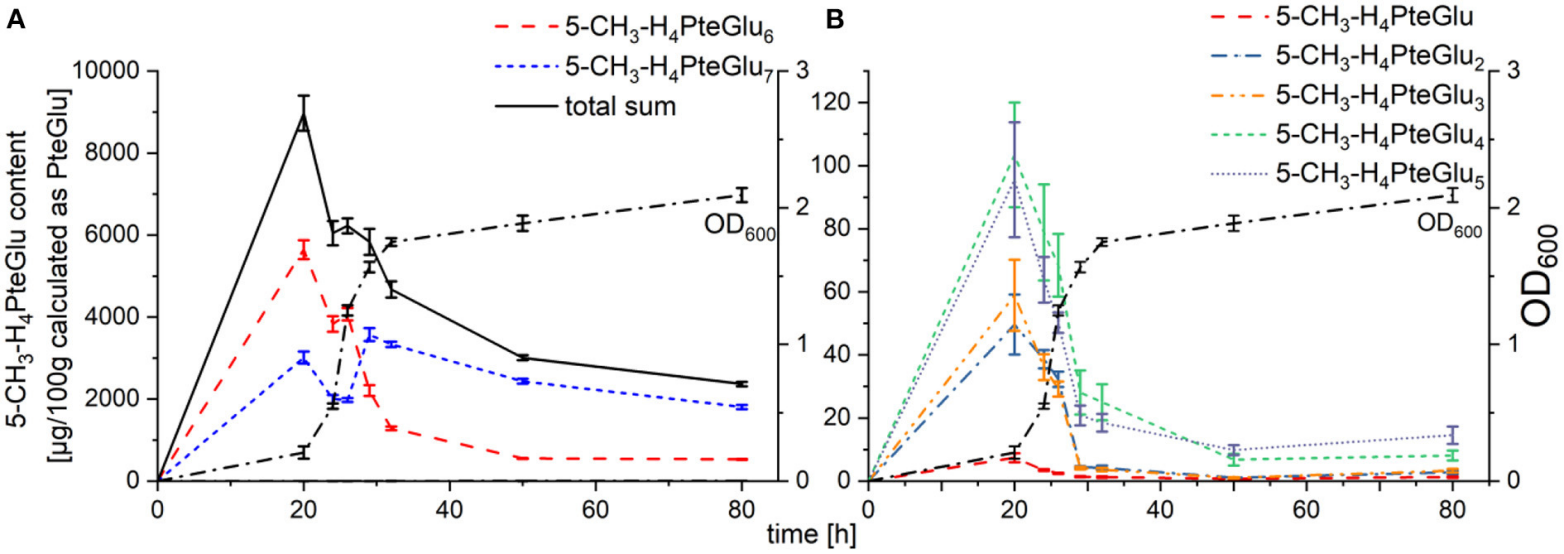
Folate contents of the polyglutamates 5-CH_3_-H_4_PteGlu_1 − 7_ in TUM68^®^ yeast during cultivation, calculated as μg/100 g PteGlu; with **(A)** main polyglutamates 5-CH_3_-H_4_PteGlu_6_ (red), 5-CH_3_-H_4_PteGlu_7_ (blue), and the total sum of 5-CH_3_-H_4_PteGlu_1 − 7_ (black) in comparison to the optical density (OD_600_, dotted line), **(B)** minor polyglutamates 5-CH_3_-H_4_folate (red), 5-CH_3_-H_4_PteGlu_2_ (blue), 5-CH_3_-H_4_PteGlu_3_ (orange), 5-CH_3_-H_4_PteGlu_4_ (green), and 5-CH_3_-H_4_PteGlu_5_ (purple) in comparison to the optical density (OD_600_, dotted line). Data are expressed as mean values ± confidence limits.

Polyglutamate contents were quantifiable with our SIDA method for the polyglutamates 5-CH_3_-H_4_PteGlu_1 − 7_. As shown in Figure 3A, main vitamers identifiable were 5-CH_3_-H_4_PteGlu_6_ and 5-CH_3_-H_4_PteGlu_7_. Furthermore, the octaglutamate 5-CH_3_-H_4_PteGlu_8_ (shown in Supplementary Figure 10) was observable, yet not quantifiable with the SIDA method applied. The sum of both main polyglutamates, 5-CH_3_-H_4_PteGlu_6_ and 5-CH_3_-H_4_PteGlu_7_, accounted for 98 ± 2% of the total 5-CH_3_-H_4_PteGlu_n_ sum throughout the whole cultivation process. 5-CH_3_-H_4_PteGlu_1 − 5_ (shown in Figure 3B) had only minor impact on the total 5-CH_3_-H_4_folate content. Worth mentioning is the change of the main polyglutamate between 26 h and 29 h. While two thirds of the polyglutamates were present in the form of 5-CH_3_-H_4_PteGlu_6_ from 20 h to 26 h, this dropped to one third for a cultivation time > 29 h. 5-CH_3_-H_4_PteGlu_8_, however, increased until 50 h of cultivation, yet never reached the levels (determined as mean peak area of all injected replicates in Supplementary Figure 10) of 5-CH_3_-H_4_PteGlu_6_ and 5-CH_3_-H_4_PteGlu_7_, respectively.

### Profiling of folate metabolites during yeast cultivation by UHPLC-Q-ToF-MS

**Figure 4:**
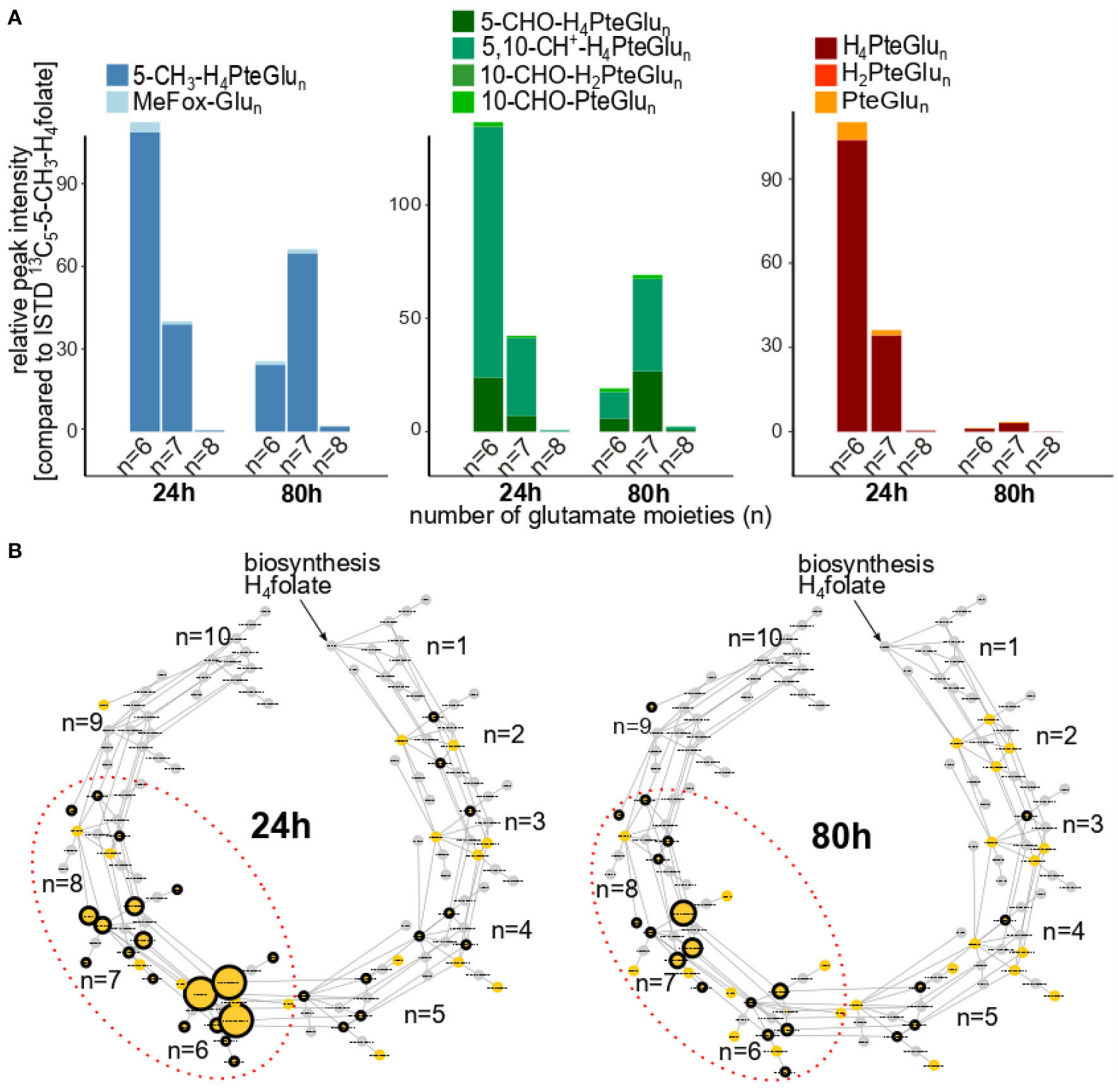
Profiling of folate metabolites in cultivated TUM68^®^ yeast after 24 and 80 h of cultivation time analyzed by UHPLC-Q-ToF-MS; with **(A)** detailed distribution of the main polyglutamates (*n* = 6–8) depicted as relative peak intensity compared to the added internal standard (ISTD) [^13^C_5_]-5-CH_3_-H_4_folate; with methylated polyglutamates 5-CH_3_-H_4_PteGlu_6 − 8_ (dark blue) and their corresponding oxidation products MeFox-Glu_6_-_8_ (light blue); formylated polyglutamates 5-CHO-H_4_PteGlu_6 − 8_ (dark green) and 5,10-CH^+^-H_4_PteGlu_6 − 8_ (leaf green) and their corresponding oxidation products 10-CHO-H_2_PteGlu_6 − 8_ and 10-CHO-PteGlu_6 − 8_ (lighter greens); unsubstituted polyglutamates H_4_PteGlu_6 − 8_ (dark red) and their corresponding oxidation products H_2_PteGlu_6 − 8_ (red) and PteGlu_6 − 8_ (orange), **(B)** folate C_1_ polyglutamate metabolism starting at H_4_folate and additional incorporation of one glutamate moiety for each subgroup represented by different indices n defining the length of the polyglutamate tail. Different nodes represent the diversity of folate vitamers in different polyglutamate states. Edges represent enzymatic transformations within the cell between those polyglutamates. Nodes shown in yellow could be determined on MS^1^ level, whereas nodes highlighted by an additional black circle were further identified on MS^2^ level. The size of the nodes displays the relative peak intensity as mean of all replicates measured.

To investigate folate metabolites in cultured yeast in more depth, we analyzed the samples by means of ultra-high pressure liquid chromatography (UHPLC) Q-ToF-MS. Generated MS-features were filtered for the respective chromatographic behavior known by the injection of folate standards. Only features eluting within the retention time window of folates (±0.2 min) were kept as described in an earlier publication (16). The profiling of folate metabolites on different polyglutamate levels revealed the occurrence of glutamate moieties in the glutamate tail ranging from *n* = 2 to *n* = 8 as shown in Figure 4. In total, 45 different polyglutamates could be determined by their *m/z* (hereafter stated as MS^1^ level, yellow nodes) of which 29 could further be identified by tandem MS/MS spectra (stated as MS^2^ hereafter, black edging) for 24 h of cultivation. Twenty one folate polyglutamates were identified on MS^2^ level for 80 h of cultivation. Decreasing intensities could be observed with increase of cultivation time (represented by the node size in Figure 4A). Furthermore, a shift in the main polyglutamate form from the hexaglutamates (after 24 h of cultivation) to the heptaglutamates (after 80 h of cultivation) was observed for all vitamers regardless their substitution (methylated, formylated and unsubstituted polyglutamates shown in Figure 4B). Supplementary Figure 11 illustrates comparable transformation rates throughout the cultivation process from the hexa- to the heptaglutamates for each of the vitamer groups. The oxidative degradation products (shown in Supplementary Figure 11B), however, were characterized by slightly higher hexaglutamate proportions in comparison to their corresponding metabolic active vitamers (shown in Supplementary Figure 11A).

### Molecular networking and putative identification of unknown folates

**Figure 5:**
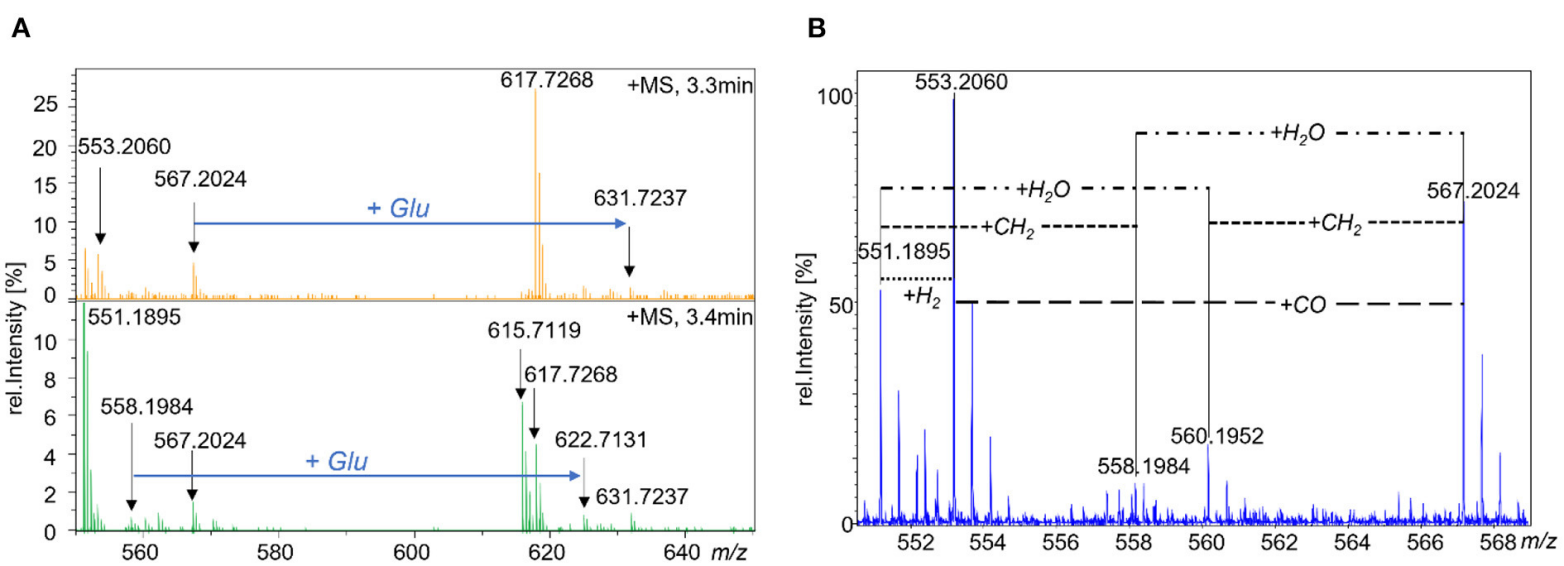
MS-spectra of cultured TUM68^®^ yeast analyzed by UPLC-Q-ToF-MS. with **(A)** MS^1^-spectra showing the putative folate vitamers of metabolite group 1 (*m/z* 558.1984 and *m/z* 622.7131) and metabolite group 2 (*m/z* 567.2024 and *m/z* 631.7237) and the mass difference between the features of each group referring to the incorporation of an additional glutamate moiety. **(B)** MS^1^ spectrum showing known and putative folate hexaglutamates with *m/z* 551.1895 = 5,10-CH^+^-H_4_PteGlu_6_, *m/z* 553.2060 = 5-CH_3_-H_4_PteGlu_6_, *m/z* 558.1984 = hexaglutamate metabolite group 1, *m/z* 560.1952 = MeFox-Glu_6_/5-CHO-H_4_PteGlu_6_, *m/z* 567.2024 = hexaglutamate metabolite group 2.

To further putatively identify folate metabolites in the yeast samples analyzed, mass spectral molecular networking was applied (Supplementary Figure 12) for each individual cultivation time point. Compounds not eluting within the respective retention time of folates (known of the profiling part) ± 0.2 min were excluded from the networks. On average, more than 60% of the clustered features referred to already known folates for each of the cultivation time points. For identification of the remaining features, we focused on common features throughout different cultivation times. Of those, we selected the nodes being observable in more than two thirds of the obtained networks. Furthermore, we checked for metabolic activity of these nodes. Therefore, we plotted the corresponding intensity of the selected nodes as a function of time during yeast cultivation. Applying those filtering functions, we aimed at only selecting those features showing metabolic significance in the context of the already known folate metabolism. Doing so, we identified four promising features which could be grouped into two subgroups based on their fragmentation behavior (shown in Supplementary Figure 13A). Within both subgroups, the corresponding MS^1^-spectra revealed a mass difference accounting for an additional incorporation of one glutamate moiety between the features of the subgroup visualized in Figure 5A. Based on the number of neutral losses observable in each of the corresponding MS/MS-spectra, the features were putatively annotated as hexa- and heptaglutamates. The exemplary visualization of the stepwise neutral loss of glutamate moieties for one of the putative hexaglutamates is shown in Supplementary Figure 13B.

For means of identification, we searched for further mass differences between the unknown compounds and already known folate vitamers which are shown in Figure 5B. The hexaglutamate of the first subgroup (*m/z* 558.1984) was linked with 5,10-CH^+^-H_4_folate (*m/z* 551.1895) by a neutral loss of 7.0089 Da referring to CH_2_ when accounting for the double charged state of the ions. Furthermore, it was linked with the hexaglutamate of the second subgroup (*m/z* 567.2024) by the addition of H_2_O (9.0050 Da). The latter revealed further mass differences accounting for the addition of CO (13.9972 Da) and CH_2_ (7.0072 Da) to 5-CH_3_-H_4_folate (*m/z* 553.2060) and MeFox-Glu_6_ (*m/z* 560.1952), respectively. As 5-CHO-H_4_folate, 10-CHO-H_4_folate and MeFox are isomeric folate forms, *m/z* 560.1952 could also represent the formyl vitamers.

The first group of metabolites was putatively identified as a C_2_ variant of the C_1_-donor 5,10-CH^+^-H_4_folate, namely 5,10-ethenyl-tetrahydrofolate (5,10-CH^+^-CH_3_-H_4_folate) due to the additional incorporation of CH_2_ into the 5,10-CH^+^-H_4_folate core structure. The existence of 5,10-CH^+^-CH_3_-H_4_folate recently has been described in *Lactobacillus reuteri* (23) and thus, its prevalence in other organisms such as yeast would be highly plausible. Compliance in the retention behavior as well as the fragmentation behavior of the C_1_- and the C_2_-metabolites observed in our studies, supported this identification further (shown in Supplementary Figure 14). Furthermore, the trend of the two polyglutamates during yeast cultivation constantly increased (visualized in Supplementary Figure 15A). Thus, this gave further hint for their identification as formyl variant as the latter had been shown to gain in content towards the end of the yeast cultivation. However, due to the huge structural similarity of folate vitamers and generally low prevalence, a further purification could not be conducted. Consequently, the identification could not further be proven.

**Figure 6:**
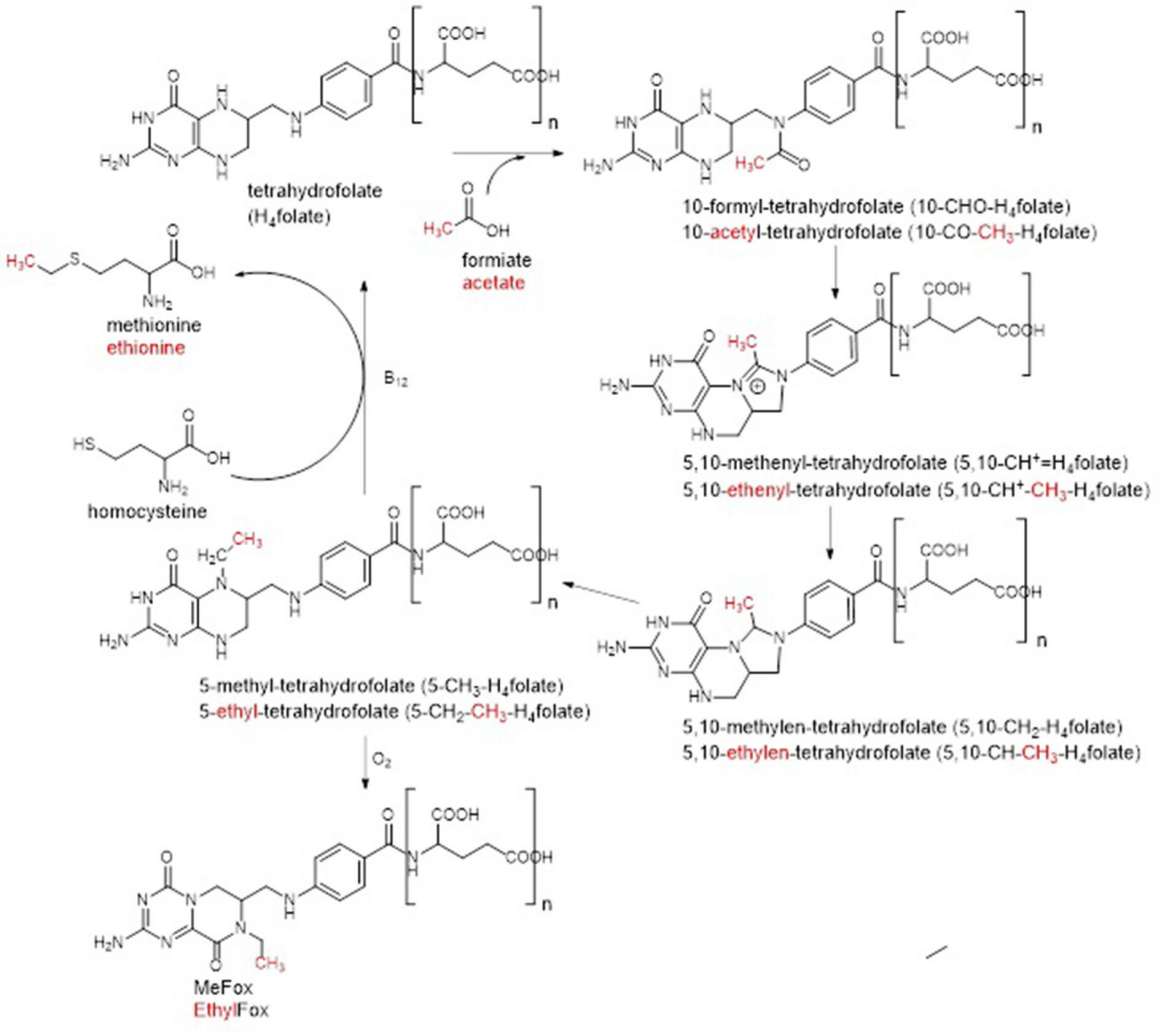
Postulation of a C_2_-metabolism in Baker's yeast by adaptation of the C_1_-metabolism. Adaptations are highlighted in red.

Several more mass differences to already known folates had been observed for the second group of metabolites enabling its annotation as C_21_H_25_N_7_O_7_ for the monoglutamate form. Hence, different theories could be postulated for the structure elucidation: (1) the metabolites of this group represent water adducts (formed during ionization of the compounds) of the first group of metabolites previously identified as 5,10-CH^+^-CH_3_-H_4_folate, (2) the metabolites are a C_2_ variant of 5-CH_3_-H_4_folate with additional incorporation of a formyl group, (3) the metabolites are a C_2_ variant of either MeFox or 5-CHO-H_4_folate with additional incorporation of a methyl group. As the metabolites of the second group showed completely different development of peak intensities during yeast cultivation compared to the first group (shown in Supplementary Figures 15A,B), theory 1 could be rejected. If they would represent different ions but refer to the same compound, they should show the same trends. Thus, according to theory (2) and (3), identification as a further C_2_-metabolite was more plausible. In accordance with the potential existence of a C_2_-metabolism shown in Figure 6, the second group of metabolites could represent the precursor of 5,10-CH^+^-CH_3_-H_4_folate within the postulated C_2_-cylce, the acetyl vitamer form 10-acetyl-tetrahydrofolate (10-CO-CH_3_-H_4_folate). The latter would be in compliance with the additional incorporation of either a formyl group into the 5-CH_3_-H_4_folate core structure [theory (2)] or an additional methyl group into the 10-CHO-H_4_folate core structure [theory (3)]. As the core structure of theory (3) could also be the isomeric form MeFox, the second group of metabolites could also be the C_2_ analog of the latter, which we name “EthylFox” throughout the rest of the manuscript. This compound would represent the oxidation product of a further C_2_-metabolite, namely 5-CH_2_-CH_3_-H_4_folate. To verify the identity of the second group of metabolites, two C_2_-metabolites, 5-CO-CH_3_-H_4_folate and EthylFox, were synthesized as mono-glutamates. Information about the synthesis and verification are provided in the Supplementary material. However, comparative measurements of the synthesized standards by UHPLC-Q-ToF-MS unraveled different retention behavior of both standards. Thus, the group of metabolites could neither be identified as 5-CO-CH_3_-H_4_folate nor as the oxidation product of 5-CH_2_-CH_3_-H_4_folate, namely EthylFox. It has to be mentioned that the 5-CO-CH_3_-H_4_folate was synthesized instead of the metabolic active 10-CO-CH_3_-H_4_folate due to higher reactivity of the position N^5^. However, the C_1_-metabolites 5- and 10-CHO-H_4_folate are hardly separable by liquid chromatography and thus also for the C_2_-analog a similar elution was assumed. Admittedly, the possibility of identification of the second metabolite group 2 as 10-CO-CH_3_-H_4_folate remains even though this is highly implausible.

## Discussion

Values for the total folate content decreased during cultivation of brewer's yeast from 13,200 μg/100 g (after 20 h) to 6,000 μg/100 g after 80 h of cultivation (Figure 2). Those values are in agreement with previous findings reporting highest total folate content in cultured yeast of 12,500 μg/100 g (24). However, these authors observed highest folate contents after 10 h of cultivation, whereas we cultured for 20 h to reach this maximum. This could be due to different yeast strains used for experimental setup. Both studies, however, revealed 5-CH_3_-H_4_folate as main vitamer with about 80% of the total folate content at maximal folate contents.

Comparing the development of 5-CH_3_-H_4_folate and H_4_folate (Figure 2A) during yeast growth in both studies, similar tendencies can be observed. In both studies, highest 5-CH_3_-H_4_folate contents occur during the respiro-fermentative phase and begin to decrease towards the end of this phase. Consequently, it can be assumed that the need for the methyl donor 5-CH_3_-H_4_folate is extraordinarily high during this growth phase. Similar observations could have been drawn for microalgae in a recent study (25). While 5-CH_3_-H_4_folate is already decreasing towards the end of the exponential growth phase in both studies, H_4_folate continues to increase until 5-CH_3_-H_4_folate reaches a relative plateau and remains constant for some time afterwards. With beginning of the stationary phase, both vitamers drop with similar rates regardless which study is considered. This highlights reduced need for C_1−_metabolism during steady state. In contrast to the studies of Hjortmo et al. (24), we could quantify further folate vitamers which was not possible with the HPLC-fluorescence method applied by the latter authors. All formyl folates analyzed as the sum of 5-CHO-H_4_folate showed the same trends compared to H_4_folate but continued to increase throughout the whole cultivation process. In contrast to the other formyl forms, 5-CHO-H_4_folate has been reported to be the only stable yet metabolic inactive formyl folate in the organism (26). Thus, it can be hypothesized, that excess folate coenzymes (mostly in the form of 5-CH_3_-H_4_folate) are being stored in form of 5-CHO-H_4_folate to easily be reactivated into 5,10-CH^+^-H_4_folate by methenyltetrahydrofolate synthetase (MTHFS). Striking was the fact that the sum of 5-CH_3_-H_4_folate and 5-CHO-H_4_folate constantly remained at 84% of the total folate content throughout the whole cultivation process further supporting this hypothesis.

Of the oxidation products which we could determine with our methods, PteGlu and 10-CHO-PteGlu (Figure 2B) showed the same tendencies compared to their metabolic active equivalents H_4_folate and 5-CHO-H_4_folate, respectively. Solely MeFox appears to rather follow the development of 5-CH_3_-H_4_PteGlu_7_ (Figure 3A) than the development of total 5-CH_3_-H_4_folate. Thus, it can be suspected that the heptaglutamate form accounts for the preferred folate polyglutamate form in yeasts and thus is more prone to oxidation reactions. Furthermore, the shift of the main polyglutamate from the hexa- to the heptaglutamate occurred during 29 and 32 h of cultivation for each of the polyglutamates analyzed by UHPLC-Q-ToF-MS (Supplementary Figure 3A). This time point represents the beginning of the stationary growth phase which is also characterized by decreasing need for folates in general. It is known that longer polyglutamates show higher affinity towards folate binding proteins (FBP) but reduce the activity of the latter (27). Consequently, it can be suspected that hexaglutamates reduce the activity of FBPs tremendously. Only a reduced number of folate monoglutamates competing with these hexaglutamates to be bound and metabolized by FBPs, enables a further elongation of the hexaglutamate tail. The reasons for the further elongation, however, are still unknown.

At the end of the exponential growth phase, oxidative degradation products were affected similarly by this shift in the polyglutamate tail length. However, ratios between hexa- and heptaglutamate form of these degradation products (shown in Supplementary Figure 3B) did not shift towards the heptaglutamate form to the same extent compared to the metabolic active vitamer forms. Three reasons for this observation are plausible: (1) longer metabolic active polyglutamates tend to be more stable (28) and a relatively higher proportion of hexaglutamates is being oxidized. (2) with elongation of the polyglutamate tail, metabolic inactive polyglutamates tend to be less stable (28) once having been oxidized and are more susceptible towards further oxidation reactions. (3) the observed results are the results of generally low abundancies of oxidation products compared to the corresponding active folate vitamers in combination with reduced response of longer polyglutamates during mass spectrometric analysis. Theory (1) is likely to be rejected as total MeFox contents were mostly deriving from 5-CH_3_-H_4_PteGlu_7_ contents which seemed to be less stable compared to the hexaglutamate form. Theories (2) and (3) are somewhat difficult to prove. However, it is more likely that both theories are contributing to the observed experimental results.

To identify similarities between obtained MS/MS spectra acquired by UHPLC Q-ToF-MS, molecular networking was performed (17). MN revealed several features mostly referring to the main occuring hexa- and heptaglutamate derivatives. Clustering features consisted up to >60% of known folate vitamers. Further known degradation products than the above mentioned, quantified oxidation products were not detected within the MS^1^ features. Detailed analysis of unknown but promising features throughout the whole sample set unraveled typical mass differences of CO, CH_2_, and H_2_O between already known and putatively as folate vitamer annotated species. The appearance of mass differences corresponding to the addition of a further methyl- or formyl group suggested the hypothesis of folate vitamers not only serving as C_1_- but also as C_2_-donors. Within the postulated C_2_-metabolism, only 5,10-CH^+^-CH_3_-H_4_folate could be found yet not finally identified as hexa- and heptaglutamate. A further group of compounds consisting of hexa- and heptaglutamates could neither be identified as 5-CO-CH_3_-H_4_folate nor as the oxidation product of 5-CH_2_-CH_3_-H_4_folate, namely EthylFox. Solely, an annotation as C_21_H_25_N_7_O_7_ could be made for the corresponding mono-glutamate. Thus, this class of compounds requires further approaches to identify its structure. Any further C_2−_reaction partners such as 5,10-CH_2_-CH_3_-H_4_folate or 5-CH_2_-CH_3_-H_4_folate could also not be found within the MS^1^features remaining the C_2_-metabolism questionable.

The detected 5,10-CH^+^-CH_3_-H_4_folate could have further origins other than the already discussed acetate. In accordance with the folate metabolism in yeasts, 5,10-CH^+^-H_4_folate is produced via three different pathways: (1) FTHFS (formyltetrahydrofolate synthetase) transfers formiate to the coenzyme H_4_folate forming 10-acetyl-tetrahydrofolate which will subsequently be metabolized into 5,10-CH^+^-H_4_folate, (2) SHMT (serine hydroxymethyl transferase) converts serine and H_4_folate into 5,10-CH_2_-H_4_folate which will subsequently be transformed into 5,10-CH^+^-H_4_folate, (3) the GCS (glycine cleavage systems) metabolizes H_4_folate and glycine yielding 5,10-CH_2_-H_4_folate which is further oxidized to 5,10-CH^+^-H_4_folate. In analogy, 5,10-CH^+^-CH_3_-H_4_folate could be traced back to acetate, homoserine, and alanine. Acetate occurs in yeasts ubiquitously as it is a by-product of alcoholic fermentation (29). Homoserine represents an intermediate of the synthesis of methionine which itself is involved in methylation reactions within the organism as well as the regeneration of H_4_folate (30, 31). Another key player in the folate metabolism, namely glutamate, is the product of the transamination of alanine and transfer of its amine function onto α-ketoglutaric acid yielding pyruvate (32). Consequently, all three precursors are involved in the metabolism of yeast with homoserine and alanine in the broad sense even being involved in the folate metabolism. Which one of the precursors does indeed account for the incorporation of the C_2_-moiety into the folate metabolism of yeasts needs to be addressed in future studies. Furthermore, more proof for the existence of the C_2_-metabolism needs to be found as well as its importance and function within the metabolism of yeasts. Last, the structure of the compound class annotated as C_21_H_25_N_7_O_7_ in the respective monoglutamate form needs to be elucidated. Therefore, different foodstuffs need to be screened to identify organisms with high concentrations of this compound which could then be used for further purification and identification.

## Conclusion

We could show that 5-CH_3_-H_4_folate and H_4_folate monoglutamates increased until a cultivation time of 20 h and 26 h, respectively. 5-CHO-H_4_folate, however, increased throughout the whole cultivation process analyzed. Interestingly, the sum of 5-CH_3_-H_4_folate and 5-CHO-H_4_folate in relation to the total folate content remained constant from 20 h to 80 h, both vitamers were quantified up to the heptaglutamate. 5,10-CH^+^-CH_3_-H_4_folate could be found as hexa- and heptaglutamate but not finally identified as such. A further folate like group of metabolites was found as hexa- and heptaglutamate which according to the sum formulae obtained could also belong to a potential C_2_-metabolism. The latter group of folates could neither be identified as 5-acetyl-tetrahydrofolate nor as the oxidation product of 5-ethyl-tetrahydrofolate, here named as EthylFox. To identify the unknown group of folate metabolites, further studies need to be conducted.

## Data availability statement

The original contributions presented in the study are included in the article/Supplementary material, further inquiries can be directed to the corresponding authors.

## Author contributions

Conceptualization: LSc, PS-K, and MR. Methodology: LSc, DW, NW, FS, and LSt. Data curation: LSc and DW. Writing: LSc. Review and editing: PS-K and MR. Funding: MR. All authors contributed to the article and approved the submitted version.

## Funding

We thank the TUM Open Access Publication Fund and the TUM School of Life Sciences for supporting the green open access publication of this study.

## Conflict of interest

The authors declare that the research was conducted in the absence of any commercial or financial relationships that could be construed as a potential conflict of interest.

## Publisher's note

All claims expressed in this article are solely those of the authors and do not necessarily represent those of their affiliated organizations, or those of the publisher, the editors and the reviewers. Any product that may be evaluated in this article, or claim that may be made by its manufacturer, is not guaranteed or endorsed by the publisher.
