# Supplementary material for: Metabolic folate profiling as a function of time during cultivation suggests potential C2-metabolism in Saccharomyces cerevisiae
Source: Front Nutr. 2022 Oct 19;9:984094. doi: 10.3389/fnut.2022.984094 (PMC9626864; doi:10.3389/fnut.2022.984094)
Supplement: Supplementary file 1 [file Data_Sheet_1.docx]

Supplementary Material

# Supplementary Data

## Cultivation and sampling of brewer’s yeast *Saccharomyces cerevisiae* (LeoBavaricus TUM68^®^)

YPD medium (2% yeast extract, 1% peptone, 2% dextrose) was used to start cultivation. Synthetical minimal medium used for experiments was prepared as described elsewhere [1] with some minor modifications. The chemicals listed in **Supplementary Table 1** were dissolved in 1 liter of distilled water. The solution was autoclaved at 121 °C for 20 min, vitamins (4 µg D-biotin, 500 µg calcium pantothenate, 500 µg nicotinic acid, 500 µg pyridoxin hydrochloride, 250 µg riboflavin, and 500 µg thiamine hydrochloride) were dissolved in 3 mL water, filter sterilized, and added to the medium before use. PBS buffer used for washing consisted of 8 g/L NaCl, 0.2 g/L KCl, 1.42g/L Na_2_HPO_4_, and 0.27 g/L K_2_HPO_4_.

An inoculation loop of an agar slant culture was transferred into 10 mL YPD medium and incubated for 48 h at 30 °C on a shaker (150 rpm). 100 µL was then inoculated into 10 mL minimal medium and 100 µL of those subsequently inoculated into 10 mL fresh minimal medium. After incubation for 72 h at 30 °C on a shaker (150 rpm), 500 mL of fresh minimal medium was transferred into Erlenmeyer flasks with a total volume of 1000 mL. 0.1% of the grown preculture at a starting optical density (OD_600_) of 0.15 were used for inoculation. Incubation was performed at 30 °C on a shaker (150 rpm). Incubation time was varied between 20 and 80 hours before yeast cells were harvested. Before harvest, optical density of the cultures was determined. A batch of 6 Erlenmeyer flasks was combined to give one homogeneous culture for each time point. Harvest was performed by centrifugation (4000 rpm, 4 °C) for 5 min. The cells were three times washed with 30 mL PBS buffer. The cell pellet was frozen and lyophilized thereafter. Lyophilized cells were homogenized using a pestle and mortar and stored at -20 °C until folate extraction and analysis.

## Quantification of 5-CH_3_-H_4_PteGlu_1-7_

For the development of a quantification method for the 5-CH_3_-H_4_folate polyglutamates, the corresponding standards 5-CH_3_-H_4_PteGlu_2-7_ had to be synthesized. These were then used to generate matrix matched response functions to enable a quantification by means of LC‑MS/MS.

### Synthesis of 5-CH_3_-H_4_PteGlu_2-7_

5-CH_3_-H_4_PteGlu_2-7_ were synthesized from the folate standards PteGlu_2-7_ which were bought from Schircks Laboratories (Jona, Switzerland). The methylation process was performed as described in section 1.2.2.1 according to the procedure by Blair and Saunders and Ndaw et al. [4, 5] with minor modifications. Deviating from section 1.2.2.1, 450 mg of NaBH_4_ were used. Furthermore, formaldehyde instead of acetaldehyde was used to yield the methylation product. In total, 1 µmol of each folic acid polyglutamate was methylated.

### Determination of the concentration of synthesized 5-CH_3_-H_4_PteGlu_2-7_ by HPLC-DAD

Concentrations of the synthesized 5-CH_3_-H_4_folate polyglutamate standards were determined by means of HPLC-DAD using a response function of 5-CH_3_-H_4_folate and assuming the same absorption coefficient at 290nm. The standards were separated on a C18 column (C18 EC, 250 × 3 mm, 5 μm, 100 Å, Macherey-Nagel, Düren, Germany). 50 mM phosphate buffer, pH 4.6 and acetonitrile served as mobile phases A and B, respectively. The gradient was as follows: in 15 min from 0% B to 10% B, increase to 20% B within 5 min, isocratic hold at 20% B for 5 min, decrease to 0% B within 2 min, equilibration at 0% B for 8 min. The injection volume was 50 µL, the flow rate was 0.6 mL/min.

### Generation of polyglutamate-free yeast

Polyglutamate-free yeast matrix was obtained by treating commercial baker’s yeast as follows. Freeze-dried and thoroughly homogenized yeast samples were mixed with extraction buffer (phosphate buffer 100 mM, pH 5 with addition of 1% ascorbic acid and 0.1% dithiothreitol), cooked for 1 h at 100 °C and incubated overnight with chicken pancreas and rat serum to deconjugate folate polyglutamates into the respective monoglutamates. The next day the samples were cooked again for 10 minutes at 100°C and after addition of activated charcoal, samples were stirred on ice for another 2 hours to bind the generated monoglutamates. The samples were vortexed for 15 minutes, cooked again for 10 minutes and centrifuged (4000 rpm, 20 minutes, 4°C) after adding 10 mL of acetonitrile. The activated charcoal was membrane filtered (0.2 µm, 13 mm) before adding the standards.

### Determination of matrix matched response functions for 5-CH_3_-H_4_PteGlu_2-7_

Polyglutamate-free yeast was spiked with different amounts of the synthesized standards 5‑CH_3_‑H_4_PteGlu_2-7_ and a constant amount of the internal standard [^13^C_5_]-5-CH_3_-H_4_folate. Sample preparation is described in section 1.3.5. Solely, Strata strong anion exchange (SAX) cartridges (quaternary amine) with a bed volume of 2 g (12 mL) were used for purification. Analysis by means of LC-MS/MS is described in detail in section 1.3.6. Matrix matched response functions were obtained by plotting the amount of analyte to internal standard against the concentration of analyte to internal standard. Obtained response functions were subjected to a linearity test according to Mandel [7].

### Sample preparation for the quantification of 5-CH_3_-H_4_PteGlu_1-7_

Sample preparation for the quantification of 5-CH_3_-H_4_PteGlu_1-7_ was as described in a previous publication [8]. Solely, 75 mg (for the determination of 5-CH_3_-H_4_-PteGlu_6/7_) and 150 mg (for the determination of 5-CH_3_-H_4_-PteGlu_1-5_) of the cultured yeast sample were used for sample analysis. Furthermore, samples were spiked with the internal standard [^13^C_5_]-5-CH_3_-H_4_folate after equilibration with extraction buffer (phosphate buffer 100 mM, pH 5 with addition of 1% ascorbic acid and 0.1% dithiothreitol) and equilibrated for another 15 min. In terms of purification by solid-phase extraction, the concentration of NaCl was increased to 10% in the elution buffer to enable complete elution from the cartridge material. For 5-CH_3_-H_4_PteGlu_1-5_, 6 mL of elution buffer were used whereas for 5-CH_3_-H_4_PteGlu_6/7_, 8 mL of elution buffer were used.

### Determination of the 5-CH_3_-H_4_PteGlu_1-7_ content by LC-MS/MS

Quantification of 5-CH_3_-H_4_PteGlu_2-7_ was performed by LC-MS/MS after chromatographic separation on C18 material (YMC Pack Pro C18, S-3 µm, 12 nm, 150 x 3 mm ID, Dinslaken, Germany). Water and acetonitrile acidified with 1% formic acid each, served as mobile phases A and B, respectively. The gradient composition was as follows: 2 min isocratic hold at 5% B, increase to 10% B within 6 min, 3 min isocratic hold at 10% B, increase to 15% B within 4 min followed by an increase to 90% B within 2 min, 2 min isocratic hold at 90% B, within 2 min to 5% B, equilibration at 5% B for 5 min. The column temperature was set to 30 °C, the injection volume was 20 µL. Details on the MRM events can be found in **Supplementary Table 5**.

## Profiling of folate polyglutamates applying UHPLC-Q-ToF-MS

Yeast extracts were analzyed for profiling studies on a Waters Acquity UHPLC System (Waters, Eschborn, Germany), coupled to a Bruker maXis^TM^ UHR-ToF-MS with an Apollo II ESI Source (Bruker Daltonics, Bremen, Germany). Separation was performed on a modified C18 column (Restek Raptor^TM^, Bad Homburg, Germany, ARC-18, 1.8 µm, 100 x 2.1 mm). 1% formic acid in water (giving pH 2) and 1% formic acid in acetonitrile were used as mobile phases A and B, respectively. The flow rate was set to 0.3 mL/min. The column temperature was set to 30 °C and 15 µL of sample were injected by full loop. Separation was achieved using the following gradient: 3.7 min pre-run time at 3% B, 1 min hold at 3% B, increase to 10% B within 1.25 min, hold at 10% B for 1.25 min, increase to 50% B within 3 min, 0.5 min hold at 50% B, increase to 99.9% B within 0.3 min, 1.7 min hold at 99.9% B, decrease to 3% B within 0.3 min.

Fragmentation of the three most abundant peaks per scan was enabled applying data dependent acquisition mode (DDA). The ESI parameters were set to the following conditions: nitrogen flow rate of 10 L/min, dry heater of 200 °C, nebulizer pressure of 2.0 bar and capillary voltage of 4500 V. Data were acquired with an acquisition rate of 5 Hz at a mass range from *m/z* 50 to *m/z* 1500. Calibration of the mass spectrometer was performed by injecting ESI-L Low Concentration Tuning Mix (Agilent, Santa Clara, CA, USA). Internal calibration of the obtained mass spectral data was achieved by injecting ESI-L Low Concentration Tuning Mix (1:4 diluted in acetonitrile) in the first 0.3 min of each LC-MS run.

## Synthesis, purification and characterization of different C_2_-metabolites

To identify features with a behavior similar to that of folate vitamers, the C_2_-metabolites 5-acetyl-tetrahydrofolate (5-CO-CH_3_-H_4_folate) and 5-ethyl-tetrahydrofolate (5-CH_2_-CH_3_-H_4_folate) were synthesized. The latter was further oxidized to EthylFox. 5-CO-CH_3_-H_4_folate and EthylFox were characterized by means of nuclear magnetic resonance (NMR) spectroscopy and ultra-high pressure liquid chromatography quadrupole time of flight mass spectrometry (UHPLC-Q-ToF-MS).

### 5-acetyl-tetrahydrofolate (5-CO-CH_3_-H_4_folate)

The C_2_-metabolite 5-CO-CH_3_-H_4_folate was synthesized from H_4_folate, purified and further characterized by 1D and 2D NMR. Retention time information was generated applying UHPLC-Q-ToF-MS.

#### Synthesis and purification

The standard 5-CO-CH_3_-H_4_folate (**Supplementary Figure 1**) was synthesized from H_4_folate according to the procedure described by Moran et al. [2]. Solely, formic acid was replaced by glacial acetic acid to yield the acetyl instead of the formyl vitamer. In brief, 4 mg of H_4_folate were dissolved in 10 mL phosphate buffer (50 mM, pH 3.6). After addition of 100 mg EDC (1-ethyl-3-(3-dimethylaminopropyl)carbodiimide) and 120 µL glacial acetic acid, the solution was stirred for 30 min at 80 °C. Subsequently, the solution was purified by HPLC-DAD using a C18 column (C18 EC, 250 × 3 mm, 5 μm, 100 Å, Macherey-Nagel, Düren, Germany). The gradient was as described in another publication [3] but 0.1% acetic acid were replaced by 0.1% formic acid in eluent A (water).

#### Qualitative characterization applying NMR-spectroscopy

Collected fractions of the purification by HPLC-DAD were evaporated to dryness, frozen and lyophilized. Prior to analysis by NMR on a Bruker AVIII system (500 MHz, Bruker, Rheinstetten, Germany), the substance was dissolved in 250 µL D_2_O and filled in a NMR tube. The ^1^H-shifts of the synthesized 5-CO-CH_3_-H_4_folate were mostly in accordance with the ^1^H-shifts of 5-CHO-H_4_folate (**Supplementary Figure 2**). However, the ^1^H-spectrum showed the additional existence of a methyl-group (δ_H_ 2.10 ppm). The H,C-correlated 2D NMR experiment (HMBC presented in **Supplementary Figure 3**) unraveled the connection of this methyl group to the carbonyl group (δ_C_ 174.7 ppm) in position N^5^. Further additional signals in the ^1^H-spectrum could be assigned to impurities of formic acid and methanol deriving from purification by HPLC-DAD.

#### Generation of retention time information applying UHPLC-Q-ToF-MS

5-CO-CH_3_-H_4_folate was measured applying UHPLC-Q-ToF-MS under the same conditions as the yeast samples before. However, the retention time of the standard (3.7 min) did not coincide with the retention time of the unknown compound class in hexa- and heptaglutamate form (3.3 min) as represented in **Supplementary Figure 4**. Mono- and polyglutamates should show slightly different retention behavior. However, those shifts should be within the range of ± 0.1 min. Furthermore, the monoglutamate should elute earlier than the polyglutamates from the chromatographic column. Consequently, the unknown compound class could not be identified as 5-acetyl-tetrahydrofolate.

### 5-ethyl-tetrahydrofolate (5-CH_2_-CH_3_-H_4_folate) and EthylFox

The C_2_-metabolite 5-CH_2_-CH_3_-H_4_folate was synthesized from PteGlu, purified and further oxidized to generate EthylFox. The latter was additionally purified and characterized by ^1^H NMR. Retention time information for EthylFox was generated applying UHPLC-Q-ToF-MS.

#### Synthesis and purification of 5-C_2_H_5_-H_4_folate

The standard 5-CH_2_-CH_3_-H_4_folate (**Supplementary Figure 5)** was synthesized from PteGlu according to the procedure described by Blair and Saunders [4] with adaptations based on the method of Ndaw et al. [5]. Furthermore, formaldehyde was replaced by acetaldehyde to yield the C_2_-metabolite instead of 5-CH_3_-H_4_folate. In brief, 4.4 mg of formic acid were dissolved in 15 mL phosphate buffer (100 mM, pH 7) and 45 mL Tris (Tris(hydroxymethyl)aminomethane)buffer (66 mM, pH 7). A spatula of Pd as catalyst and 2 mL octanol to avoid foaming were added. After addition of 1.5 g NaBH_4_, the mixture was equilibrated for one hour at room temperature. Adjustment of the solution to pH 7.4 by addition of acetic acid (5 M) was performed prior to the addition of 1.8 mL acetaldehyde and within 30 seconds the addition of another 1.5 g NaBH_4_. Incubation of the mixture for another hour at room temperature was performed, before the pH was lowered to pH < 1 by adding hydrochlorid acid (conc.) and equilibration of the solution for 10 min. A final addition of 1.5 g NaBH_4_ was performed after raising the pH to pH 5 by adding NaOH solution (5 M). Incubating the solution for a further hour completed the synthesis procedure.

The synthesized standard was purified by preparative HPLC on C18 material (YMC Actus, Triart C18, 150 x 20.0 mm, I.D. S-5 µm, 12 nm, YMC, Dinslaken, Germany). Water acidified with 0.1% formic acid and methanol served as mobile phases A and B, respectively. The flow rate was set to 3 mL/min, the injection volume was 1 mL. The gradient was as follows: isocratic hold at 10% B for 10 min, increase to 35% B within 8 min, isocratic hold at 35% B for 5 min, increase to 100% B within 2 min, isocratic hold at 100% B for 1 min, decrease to 10% B within 2 min, isocratic hold at 10% B for 5 min. The collected fractions were evaporated to dryness and re-dissolved in 2 mL H_2_O prior to further oxidation.

#### Synthesis and purification of EthylFox

The oxidation of 5-CH_2_-CH_3_-H_4_folate was achieved according to the procedure described by Gapski et al. [6]. The purified 5-CH_2_-CH_3_-H_4_folate was incubated with 500 µL H_2_O_2_ solution (30%) for two hours. Oxidation was stopped by the addition of sodium thiosulfate solution (100 mM). Purification was achieved by HPLC-DAD using the above mentioned method [3]. Solely, 0.1% acetic acid in mobile phase A was replaced by 0.1% formic acid.

#### Qualitative characterization of EthylFox applying NMR-spectroscopy

Collected fractions of the purified EthylFox (**Supplementary Figure 6**) were evaporated to dryness, frozen and lyophilized. Prior to analysis by NMR on a Bruker AVIII system (500 MHz, Bruker, Rheinstetten, Germany), the substance was dissolved in 250 µL D_2_O and filled in a NMR tube. The ^1^H-shifts of the synthesized EthylFox were mostly in accordance with the ^1^H-shifts of MeFox (**Supplementary Figure 7**). The ^1^H-spectrum and the H,H-correlated 2D NMR experiment (COSY presented in **Supplementary Figure 8**) of EthylFox showed an additional ethyl group (δ_H_ 1.29 ppm, 3.27 ppm, 3.95 ppm). The H,C correlated HMBC experiment showed the position of this ethyl group at position N^7^. Further additional signals in the ^1^H-spectrum could be assigned to impurities of formic acid, acetic acid, and methanol deriving from purification by HPLC-DAD.

#### Generation of retention time information applying UHPLC-Q-ToF-MS

EthylFox was measured applying UHPLC-Q-ToF-MS under the same conditions as the yeast samples before. However, the retention time of the standard (4.0 min) did not coincide with the retention time of the unknown compound class in hexa- and heptaglutamate form (3.3 min) as represented in **Supplementary Figure 4**. In accordance with assumptions already mentioned for 5‑CO-CH_3_-H_4_folate, the unknown compound class could thus also not be identified as EthylFox.

# Supplementary Figures and Tables

## Supplementary Tables

Supplementary Table 1. Composition of synthetic minimal medium used for cultivation of brewer’s yeast. Addition of a filter-sterilized vitamin solution was performed after sterilizing of the medium by autoclaving (120 °C, 20 min).

| Chemical | g/L |
| --- | --- |
| (NH_4_)_2_SO_4_ | 5.0 |
| H_3_BO_3_ | 0.0005 |
| CaCl_2_ | 0.1 |
| FeSO_4_ ∙ 7 H_2_O | 0.0004 |
| glucose | 10.0 |
| inositol | 0.002 |
| Na_2_HPO_4_ ∙ 2 H_2_O | 0.1 |
| KH_2_PO_4_ | 2 |
| KI | 0.0001 |
| CuSO_4_ | 0.00004 |
| MgSO_4_ ∙ 7 H_2_O | 1.0 |
| MnSO_4_ | 0.0004 |
| NaCl | 0.1 |
| Na_2_MoO4_∙_2 H_2_O | 0.0002 |
| ZnSO_4_ ∙ H_2_O | 0.00044 |
| D-biotin | 4 µg |
| calcium pantothenate | 500 µg |
| nicotinic acid | 500 µg |
| pyridoxin hydrochloride | 500 µg |
| riboflavin | 250 µg |
| thiamine hydrochloride | 500 µg |

Supplementary Table 2. Included *m/z* ranges into the data dependent acquisition (DDA) preference list for folate analysis by UHPLC-Q-ToF-MS.

| *m/z* range | *m/z* range continued |
| --- | --- |
| 493.62-493.72 | 622.67-622.77 |
| 552.14-552.24 | 623.66-623.76 |
| 558.15-558.25 | 625.67-625.77 |
| 559.14-559.24 | 631.67-631.77 |
| 561.15-561.25 | 687.19-687.29 |
| 567.15-567.25 | 696.19-696.29 |
| 616.67-616.77 |  |

Supplementary Table 3. Chemical shifts of 5-CO-CH_3_-H_4_folate analyzed by ^1^H-NMR spectroscopy on a Bruker AVIII system (500 MHz). -, data not available.

| position | δ ppm | integral | multiplicity | J [Hz] |
| --- | --- | --- | --- | --- |
| 2’/6’ | 7.63 | 2H | d | 8.66 |
| 3’/5’ | 6.70 | 2H | d | 8.71 |
| α | 4.34 | 1H | dd | 9.04; 4.58 |
| 7 | 3.50 | 2H | m | - |
| 6 | 3.40 | 1H | dd | 13.24; 5.00 |
| 9 | 3.30 | 1H | m | - |
| 9 | 3.16 | 1H | m | - |
| γ | 2.38 | 2H | m | - |
| β | 2.19 | 1H | m | - |
| 12 | 2.10 | 3H | s | - |
| β | 2.02 | 1H | d | 8.06 |

Supplementary Table 4. Chemical shifts of EthylFox analyzed by ^1^H-NMR spectroscopy on a Bruker AVIII system (500 MHz). -, data not available.

| position | δ ppm | integral | multiplicity | J [Hz] |
| --- | --- | --- | --- | --- |
| 2’/6’ | 7.55 | 2H | d | 9.00 |
| 3’/5’ | 6.51 | 2H | d | 8.96 |
| 5 | 4.50 | 1H | d | 14.42 |
| α | 4.42 | 1H | dd | 8.25; 4,28 |
| 6 | 4.17 | 1H | m | - |
| 11 | 3.95 | 1H | dq | 14.29; 7.22 |
| 9 | 3.86 | 1H | m | - |
| 9 | 3.54 | 1H | d | 15.57 |
| 11 | 3.27 | 1H | dq | 14.10; 7.18 |
| γ | 2.43 | 2H | qt | 15.77; 7.95 |
| β | 2.27 | 1H | dd | 11.95; 6.65 |
| β | 2.09 | 1H | dt | 16.69; 8.42 |
| 12 | 1.29 | 3H | t | 7.26 |

Supplementary Table 5. Mass transitions and parameters of 5-CH_3_-H_4_PteGlu_1-8_ for the quantification by LC-MS/MS.

| analyte MRM | precursor ion [*m/z*] | product ion [*m/z*] | dwell time [ms] | Q1 (Pre Bias)  [V] | CE  [V] | Q3 (Pre Bias)  [V] |
| --- | --- | --- | --- | --- | --- | --- |
| 5-CH_3_-H_4_PteGlu_1_ | 460.20 | 313.20 | 70.0 | -13.0 | -20.0 | -17.0 |
|  | 460.20 | 180.15 | 70.0 | -13.0 | -37.0 | -14.0 |
|  | 460.20 | 194.25 | 70.0 | -23.0 | -33.0 | -22.0 |
| 5-CH_3_-H_4_PteGlu_2_ | 589.30 | 313.20 | 70.0 | -28.0 | -25.0 | -25.0 |
|  | 589.30 | 180.15 | 70.0 | -36.0 | -65.0 | -25.0 |
| 5-CH_3_-H_4_PteGlu_3_ | 359.65 | 313.20 | 70.0 | -20.0 | -15.0 | -22.0 |
|  | 359.65 | 180.15 | 70.0 | -20.0 | -33.0 | -20.0 |
| 5-CH_3_-H_4_PteGlu_4_ | 424.15 | 313.20 | 70.0 | -15.0 | -20.0 | -21.0 |
|  | 424.15 | 180.15 | 70.0 | -15.0 | -40.0 | -13.0 |
| 5-CH_3_-H_4_PteGlu_5_ | 488.65 | 313.20 | 70.0 | -30.0 | -25.0 | -34.0 |
|  | 488.65 | 180.15 | 70.0 | -30.0 | -62.0 | -20.0 |
| 5-CH_3_-H_4_PteGlu_6_ | 553.20 | 313.20 | 70.0 | -18.0 | -20.0 | -34.0 |
|  | 553.20 | 180.15 | 70.0 | -18.0 | -45.0 | -20.0 |
| 5-CH_3_-H_4_PteGlu_7_ | 617.70 | 313.20 | 70.0 | -34.0 | -20.0 | -36.0 |
|  | 617.70 | 180.15 | 70.0 | -34.0 | -53.0 | -20.0 |
| 5-CH_3_-H_4_PteGlu_8_ | 682.20 | 313.20 | 70.0 | -12.0 | -16.0 | -21.0 |
|  | 682.20 | 180.15 | 70.0 | -12.0 | -36.0 | -13.0 |

## Supplementary Figures

Supplementary Figure 1. Chemical structure of the C_2_-metabolite 5-acetyl-tetrahydrofolate (5-CO-CH_3_-H_4_folate).


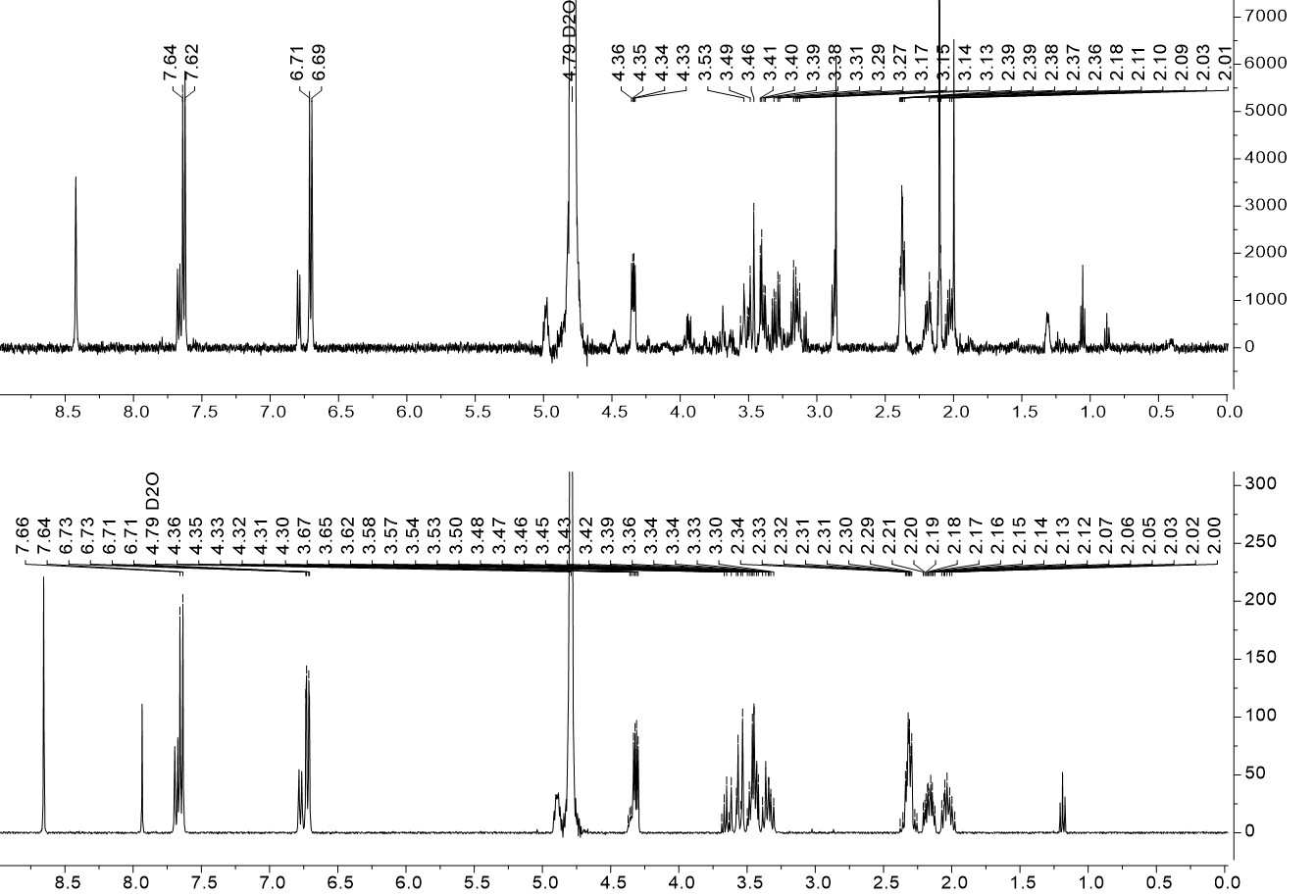


Supplementary Figure 2. ^1^H spectra of 5-CO-CH_3_-H_4_folate (top) and 5-CHO-H_4_folate (bottom) aquired at a Bruker AVIII system (500 MHz).


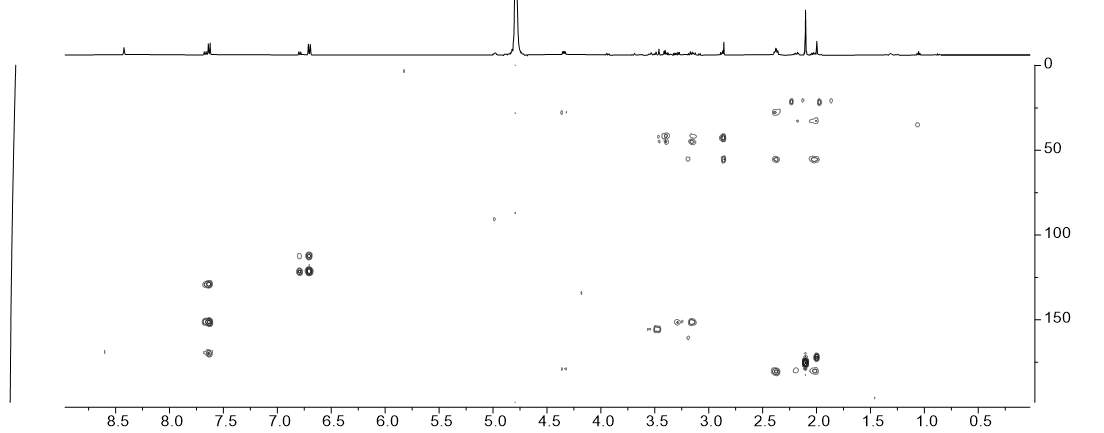


Supplementary Figure 3. ^1^H-^1^H HMBC NMR spectrum of 5-CO-CH_3_-H_4_folate.


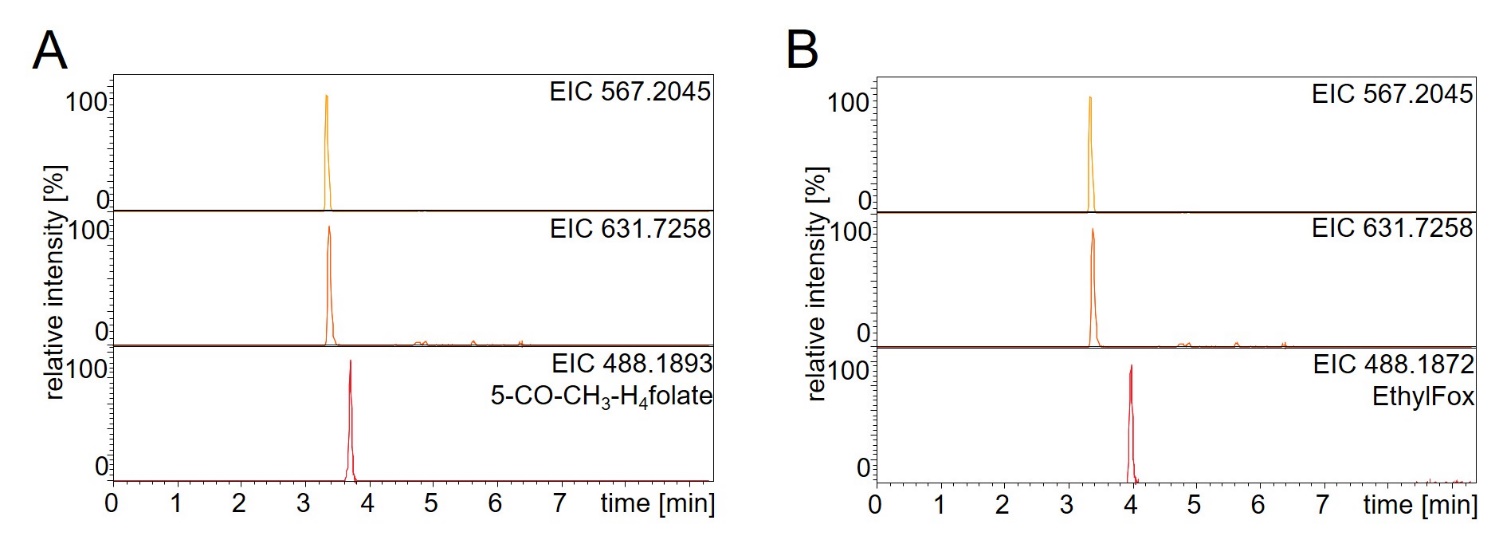


Supplementary Figure 4. Extracted ion chromatograms (EIC) of UHPLC-Q-ToF-MS analysis of C_2_-metabolites identified in cultured yeast in comparison to the synthesized standards 5-CO-CH_3_-H_4_folate (A) and EthylFox (B). No coelution of the standards with the identified MS^1^-features in the baker’s yeast samples could be observed.

Supplementary Figure 5. Chemical structure of the C_2_-metabolite 5-ethyl-tetrahydrofolate (5-CH_2_-CH_3_-H_4_folate).

Supplementary Figure 6. Chemical structure of the oxidation product EthylFox.


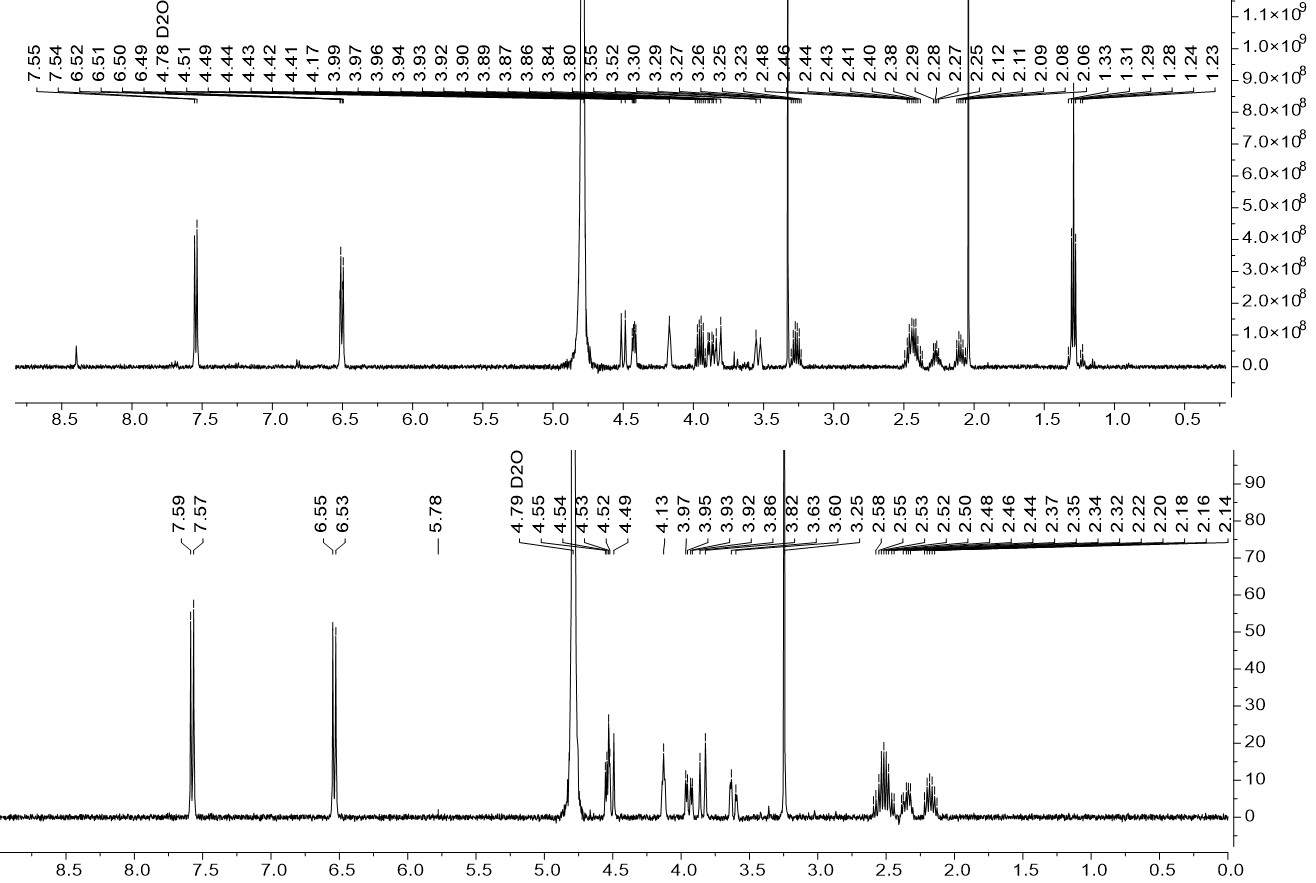


Supplementary Figure 7. ^1^H spectra of EthylFox (top) and MeFox (bottom) aquired at a Bruker AVIII system (500 MHz).


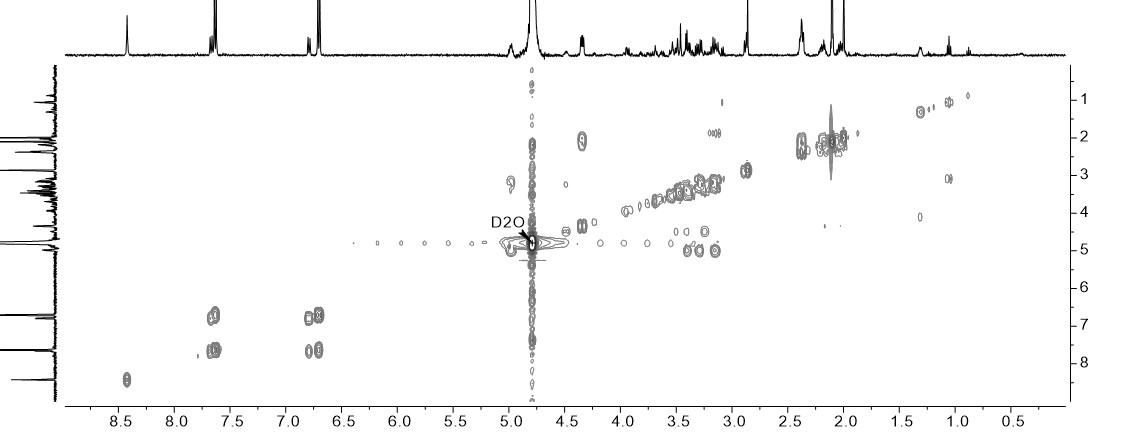


Supplementary Figure 8. ^1^H-^1^H COSY NMR spectrum of EthylFox.


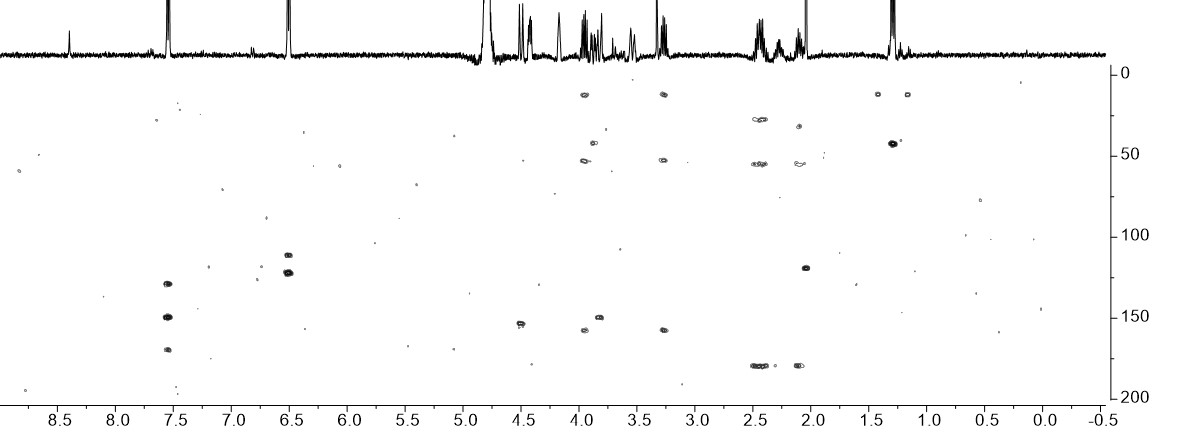


Supplementary Figure 9. ^1^H-^1^H HMBC NMR spectrum of EthylFox.


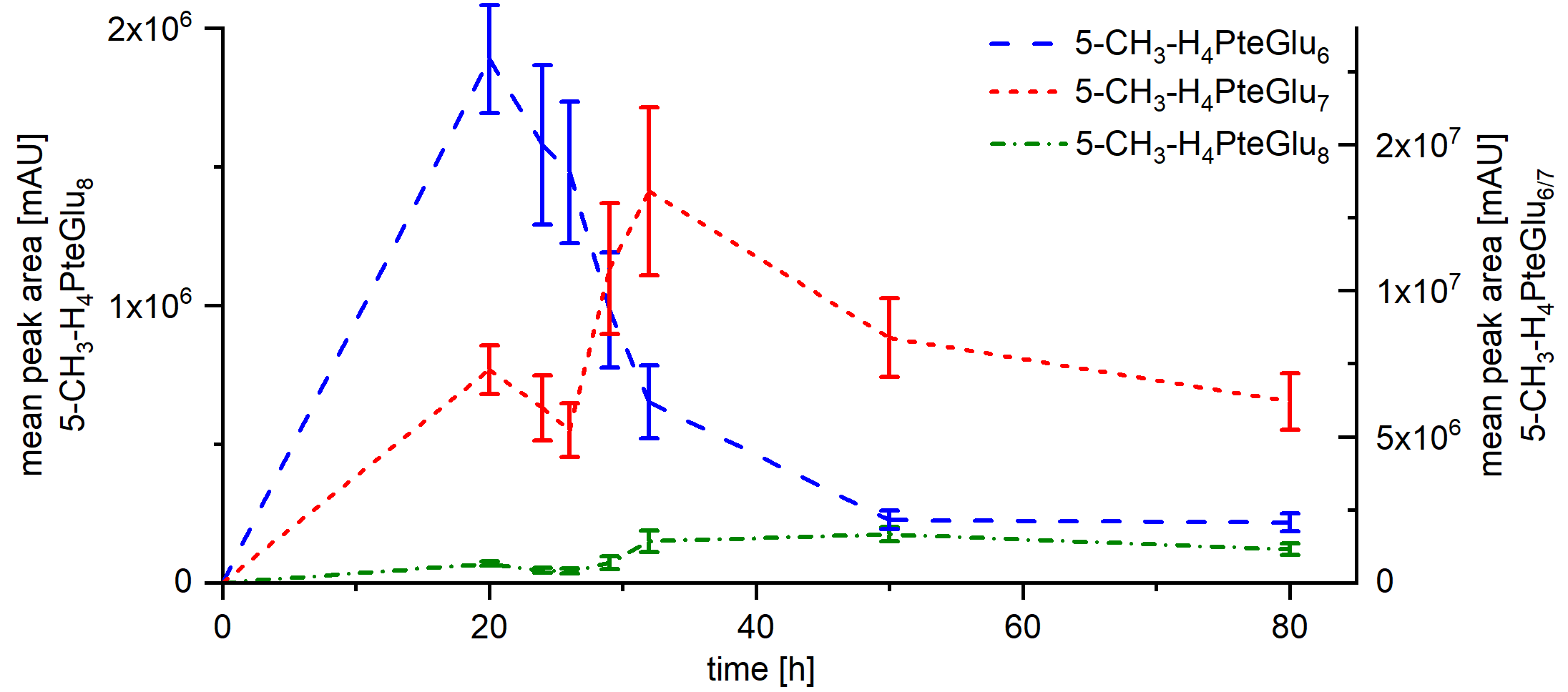


Supplementary Figure 10. Peak area of the main polyglutamates 5-CH_3_-H_4_PteGlu_6-8_ in cultivated TUM68^®^ yeast as mean of the injected replicates determined by LC-MS/MS.


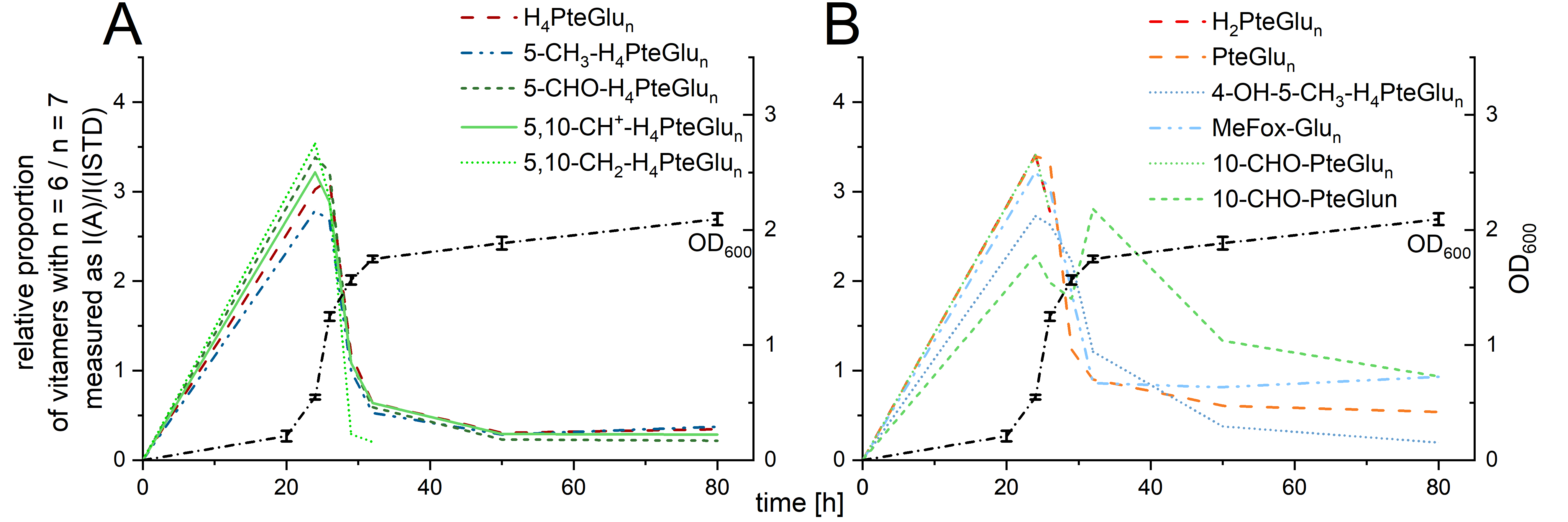


Supplementary Figure 11. Proportion of relative peak intensities of hexaglutamates relative to the heptaglutamates. Relative peak intensities were calculated in relation to the added internal standard [^13^C_5_]-5-CH_3_-H_4_folate in cultivated TUM68^®^ yeast as mean of the injected replicates determined by UHPLC-Q-ToF-MS. The growth phase was determined by measurements of the optical density at λ = 600 nm (OD_600_).


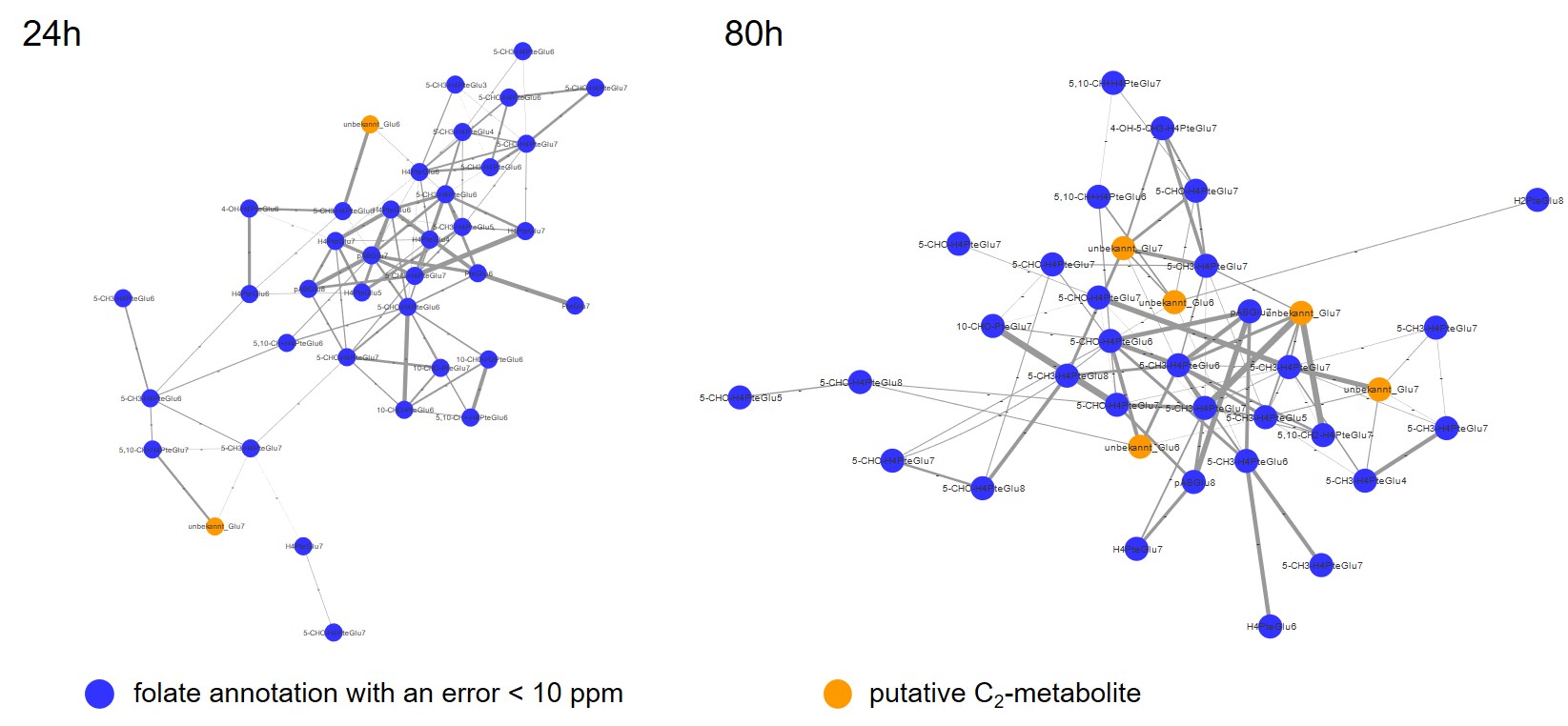


Supplementary Figure 12. Molecular networks of MS^2^ spectra referring to folates in yeast cultured for 24 h and 80 h. Molecular networks were generated by Global Natural Products Social Molecular Networking (GNPS) and exported into Cytoscape Software (https://cytoscape.org/). Received MS^2^ spectra of Q-ToF-MS measurements were used after data preprocessing by Genedata Refiner MS software. Parameters used for network generation were as follows: minimum pairs cosine: 0.6, minimum matched fragment ions: 5, minimum cluster size: 2, precursor ion tolerance: 0.02 Da, fragment ion tolerance: 0.02 Da. Different ionic compounds are depicted by the nodes while the edges (connections) represent a similarity between those compounds. The width of the edges is depicted proportional to the calculated cosine score. Features clustering in the respective folate cluster were further processed and verified in R software version 3.6.1 in combination with Compass DataAnalysis 4.3. Annotation was accepted within an annotation error < 10 ppm.


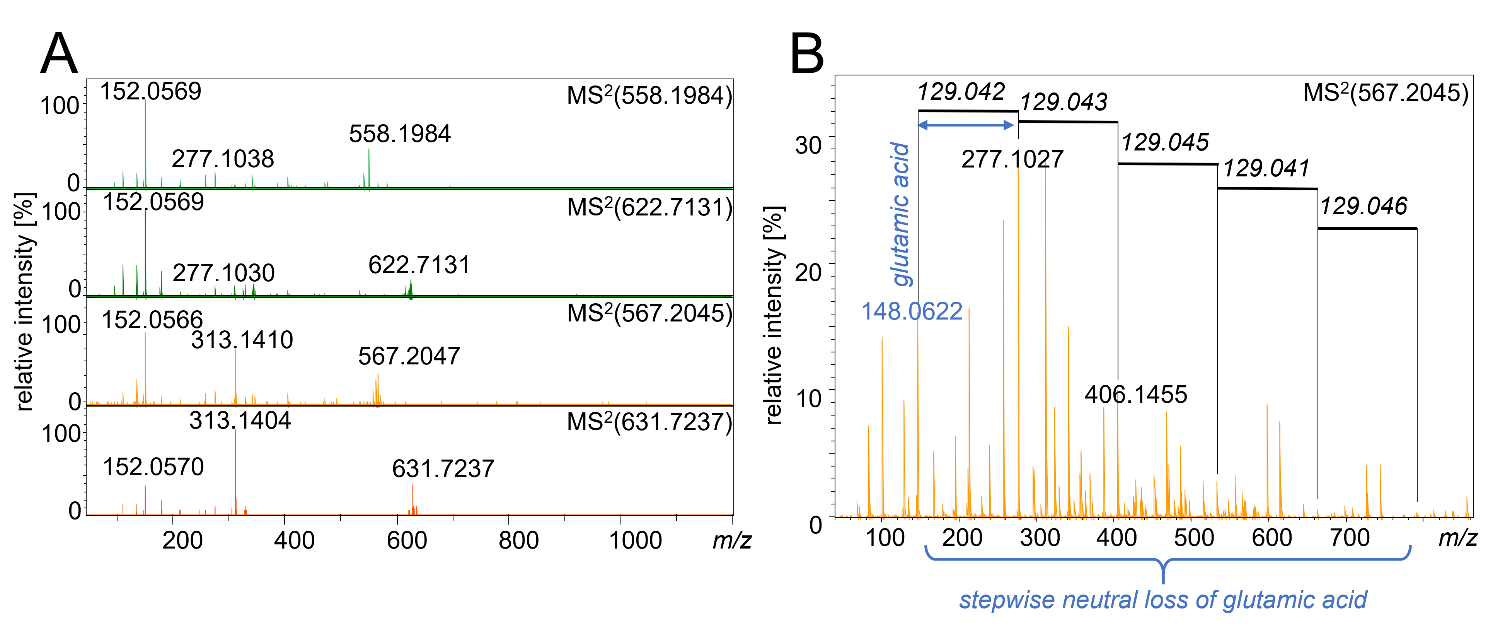


Supplementary Figure 13. Tandem MS/MS spectra of putative folate vitamers. (A) MS/MS spectra of the two groups showing the hexa- and the heptaglutamate form each with traces 1 and 2 showing group 1, and traces 3 and 4 showing group 2, respectively. (B) visualization of the stepwise neutral loss in the MS/MS spectra referring to the loss of glutamate moieties identifying the putative folates as hexa- and heptaglutamates.


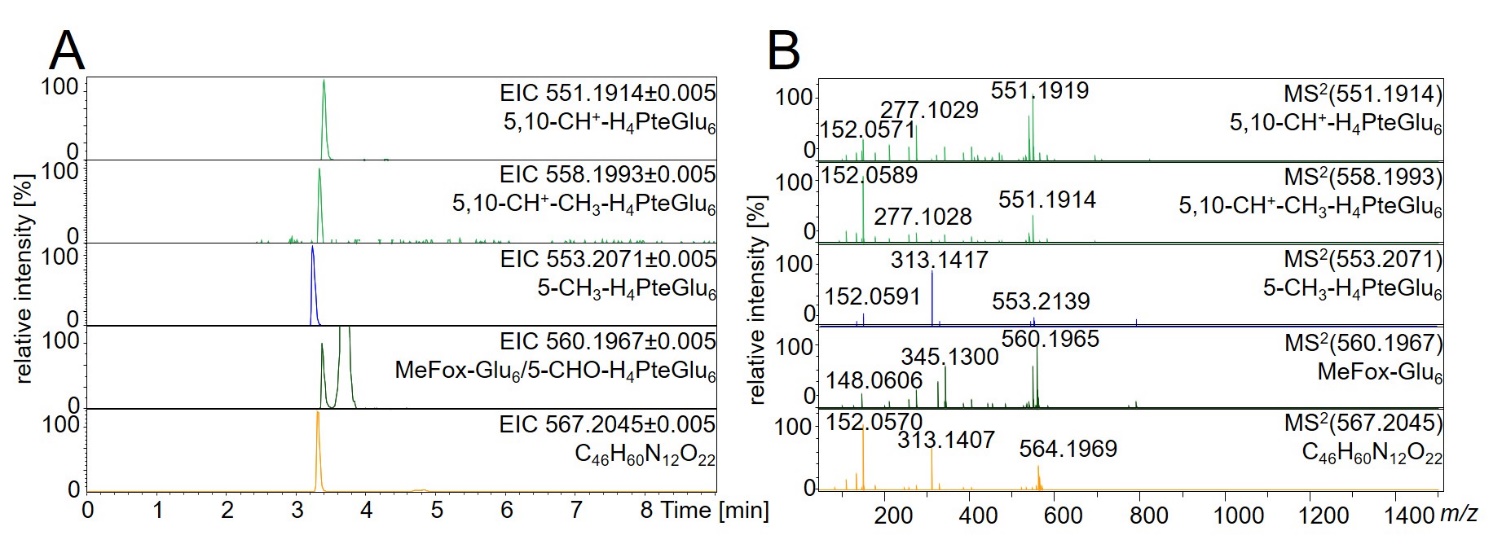


Supplementary Figure 14. Chromatographic traces of putative and already known hexaglutamates. (A) Extracted Ion Chromatograms (EIC) with an annotation error ±0.005 Da, (B) Corresponding MS/MS spectra.


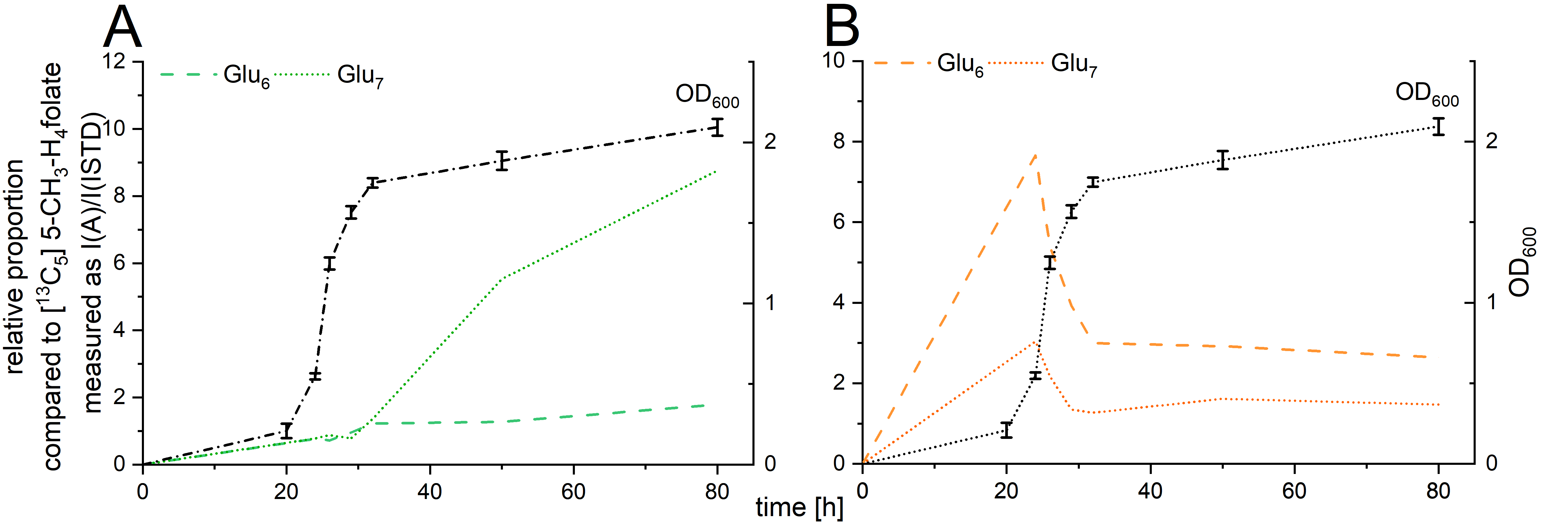


Supplementary Figure 15. Relative proportion of putative folate vitamers as function of time during the cultivation of baker’s yeast. The proportion was calculated as ratio of the peak intensities in comparison to the added internal standard (ISTD) [^13^C_5_] 5-CH_3_-H_4_folate. The growth phase was determined by measurements of the optical density at λ = 600 nm (OD_600_). (A) proportions shown for the hexa- and heptaglutamate of metabolite group 1, (B) proportions shown for hexa- and heptaglutamates of metabolite group 2.

# References

[1] Zonneveld BJM. Cheap and Simple Yeast Media. *J Microbiol Methods* (1986) 4(5-6):287-91. doi: 10.1016/0167-7012(86)90040-0.

[2] Moran RG, Colman PD. A Simple Procedure for the Synthesis of High Specific Activity Tritiated (6s)-5-Formyltetrahydrofolate. *Anal Biochem* (1982) 122(1):70-8. doi: 10.1016/0003-2697(82)90252-4.

[3] Striegel L, Chebib S, Netzel ME, Rychlik M. Improved Stable Isotope Dilution Assay for Dietary Folates Using Lc-Ms/Ms and Its Application to Strawberries. *Front chem* (2018) 6:11. doi: 10.3389/fchem.2018.00011.

[4] Blair JA, Saunders KJ. A Convenient Method for the Preparation of Dl-5-Methyltetrahydrofolic Acid (Dl-5-Methyl-5,6,7,8-Tetrahydropteroyl-L-Monoglutamic Acid). *Anal Biochem* (1970) 34(2):376-81. doi: 10.1016/0003-2697(70)90122-3.

[5] Ndaw S, Bergaentzlé M, Aoudé-Werner D, Lahély S, Hasselmann C. Determination of Folates in Foods by High-Performance Liquid Chromatography with Fluorescence Detection after Precolumn Conversion to 5-Methyltetrahydrofolates. *J Chromatogr A* (2001) 928(1):77-90. doi: 10.1016/s0021-9673(01)01129-3.

[6] Gapski GR, Whiteley JM, Huennekens FM. Hydroxylated Derivatives of 5-Methyl-5,6,7,8-Tetrahydrofolate. *Biochem* (1971) 10(15):2930-4. doi: 10.1021/bi00791a021.

[7] Mandel J, Mansfield JE. The Statistical Analysis of Experimental Data. *Phys Today* (1965) 18(9):66-8. doi: 10.1063/1.3047724.

[8] Gmelch L, Wirtz D, Witting M, Weber N, Striegel L, Schmitt-Kopplin P, et al. Comprehensive Vitamer Profiling of Folate Mono- and Polyglutamates in Baker's Yeast (Saccharomyces Cerevisiae) as a Function of Different Sample Preparation Procedures. *Metabolites* (2020) 10(8). doi: 10.3390/metabo10080301.
